# Identification of fatty acid signature to predict prognosis and guide clinical therapy in patients with ovarian cancer

**DOI:** 10.3389/fonc.2022.979565

**Published:** 2022-10-04

**Authors:** Tiefeng Cao, Jiaqi Dong, Jiaming Huang, Zihao Tang, Huimin Shen

**Affiliations:** ^1^ Department of Gynecology and Obstetrics, First Affiliated Hospital of Sun Yat-Sen University, Guangzhou, China; ^2^ Department of Oncology, First Affiliated Hospital of Sun Yat-Sen University, Guangzhou, China

**Keywords:** fatty-acid metabolism, cancer immunity, therapy response, prognosis, ovarian cancer

## Abstract

High-grade serous ovarian cancer (HGSOC) is a heterogeneous cancer characterized by high relapse rate. Approximately 80% of women are diagnosed with late-stage disease, and 15–25% of patients experience primary treatment resistance. Ovarian cancer brings tremendous suffering and is the most malignant type in all gynecologic malignancies. Metabolic reprogramming in tumor microenvironment (TME), especially fatty acid metabolism, has been identified to play a crucial role in cancer prognosis. Yet, the underlying mechanism of fatty acid metabolism on ovarian cancer progression is severely understudied. Recently, studies have demonstrated the role of fatty acid metabolism reprogramming in immune cells, but their roles on cancer cell metastasis and cancer immunotherapy response are poorly characterized. Here, we reported that the fatty acid–related genes are aberrantly varied between ovarian cancer and normal samples. Using samples in publicly databases and bio-informatic analyses with fatty acid–related genes, we disentangled that cancer cases can be classified into high- and low-risk groups related with prognosis. Furthermore, the nomogram model was constructed to predict the overall survival. Additionally, we reported that different immune cells infiltration was presented between groups, and immunotherapy response differed in two groups. Results showed that our signature may have good prediction value on immunotherapy efficacy, especially for anti–PD-1 and anti–CTLA-4. Our study systematically marked the critical association between cancer immunity in TME and fatty acid metabolism, and bridged immune phenotype and metabolism programming in tumors, thereby constructed the metabolic-related prognostic model and help to understand the underlying mechanism of immunotherapy response.

## Introduction

Tumor microenvironment (TME) plays an increasingly critical role in the pathogenesis, progression, and metastasis of multiple cancers and has great influences on patient therapy sensitivity and therapy strategies choice (1, 2). Multiple aspects and parameters of the TME may be associated with cancer prognosis and immunotherapies response (3, 4). In particular, fatty acid metabolism plays a critical influence on tumor environment and tumor immunity. In addition to providing a large amount of energy, recent studies showed that fatty acid oxidation contributes to cancer cell growth, stemness, and chemotherapy resistance. Biologically active lipid molecules during fatty acid metabolism may participate in a variety of signaling pathways in cancer cell proliferation, differentiation, metastasis, and inflammation. For example, fatty acids can enhance STAT3 palmitoylation and directly activate STAT3 in synergy with cytokine stimulation, thus promoting the tumor spheres formation and tumorigenesis (5). In addition, increased leptin and PD-1 can drive fatty acid oxidation through STAT3, inhibiting CD8^+^ T effector cell glycolysis and promoting breast tumorigenesis (6). Thus, it is critical to understand the role of fatty acid metabolism and the impact on therapy response or strategies.

Ovarian cancer is the most lethal one in gynecological cancer, and the standard mode of therapy is surgery followed by chemotherapy. Ovarian cancer is characterized by high relapse and treatment resistance rate (7), but it is considered to be the “immunogenic tumors.” In the recent years, immune therapies such as immune checkpoint blockades (ICBs) have been developed rapidly and investigated as potential maintenance treatments in ovarian cancer, including anti–PD-1 (programmed cell death protein 1), anti–PD-L1 (programmed cell death ligand 1), and anti–CTLA-4 (cytotoxic T lymphocyte-associated antigen-4) (8, 9). ICBs can attenuate the immunosuppressive signals in the TME and stimulate the antigen-presenting cells and bolster effector T cells to play key roles in the immunotherapy of ovarian cancer. But the objective response rates of single-agent checkpoint inhibitors in ovarian cancer are approximately only 6–15% (10, 11), and up to 85% of cases have resistance to ICBs. Therefore, it is urgently needed to identify the cases that could benefit from the ICBs therapy.

Recent studies on the TME, especially the fatty acid metabolism, have revealed that targeting fatty acids can promote anoikis and attenuate dissemination in ovarian cancer (12). Fatty acids generation and oxidation play a crucial role in ovarian cancer cell survival by influencing tumor immunity and immune cell infiltrates. Indeed, it remains unexplored about the critical role of fatty acid metabolism on immunotherapy response. In this study, gene expression and clinical information of 376 patients were analyzed from TCGA to comprehensively assess the pattern of fatty acid metabolism in TME. Then, a fatty acid gene-based prognostic model was constructed to divide ovarian cancer samples into high- and low-risk groups with different prognostic outcome. Additionally, we investigated the relationship between fatty acid metabolism-related model and immune cell infiltrates or immunotherapy response, showing that different immune cells infiltrated in individual TME and illustrating that this fatty acid prognostic model could distinguish ovarian cancer patients that have immunotherapy response.

## Methods

### Data collection and pre-processing

Genome data from RNA-seq (FPKM) and clinical information of 379 serous ovarian cancer samples were downloaded from TCGA (https://www.cancer.gov/about-nci/organization/ccg/research/structural-genomics/tcg), and three repeated samples were removed. Information for 88 normal ovarian samples was obtained from GTEx in xena (http://xenabrowser.net/). IMvigor210 cohort, a cohort of patients with bladder urothelial carcinoma (BLCA) treated with anti–PD-L1, was downloaded from the GEO database (https://www.ncbi.nlm.nih.gov/geo/), and it was used to analyze the relationship between risk score and immunotherapy response. GSE26712 and GSE63885 were downloaded from the GEO database as the extrinsic validation datasets. All included ovarian cancer women from TCGA and GEO datasets are pathologically diagnosed. All samples have integrity RNA-seq data, clinical information, and complete overall survival (OS) data. Gene expression level was applied with log_2_(X+1) and defined as the average value for multiple probes. All statistics were under R condition, and the “Combat” function in sva package was used to normalize gene expression distribution in different datasets. Clinical information of all datasets was shown in **Table 1**.

**Table 1:** Patient characteristics of TCGA cohort, GSE26712, and GSE63885.

	TCGA-OV-Training set	TCGA-OV-Testing set	TCGA-OV	GSE26712	GSE63885
No. with OS	224	144	368	185	75
Age (median, range)	(60,30-87)	(58,34-85)	(59,30-87)	NA	NA
Grade (%)				high-grade	
Grade 1	0	1 (0.7%)	1 (0.2%)		9 (12%)
Grade 2	27 (12.1%)	14 (9.7%)	42 (11.4%)		0 (0%)
Grade 3	193 (86.2%)	124 (86.1%)	316 (85.8%)		48 (64%)
Grade 4	0	1 (0.7%)	9 (2.6%)		18 (24%)
Unknown	4 (1.7%)	4 (2.8%)			
Stage				Late-stage	
I	0	0	0		0
II	14 (6.3%)	6 (4.2%)	20 (5.4%)		2 (2.7%)
III	176 (78.6%)	113 (78.5%)	289 (78.6%)		63 (84%)
IV	33 (14.7%)	23 (15.9%)	56 (15.2%)		10 (13.3%)
Unknown	1 (0.4%)	2 (1.4%)	3 (0.8%)		
Surgery outcome					NA
Optimal	141 (62.9%)	94 (65.2%)	235 (63.8%)	90 (48.6%)	
Suboptimal	62 (27.6%)	22 (22.9%)	95 (25.8%)	95 (51.4%)	
Unknown	21 (9.3%)	17 (11.9%)	38 (10.3%)		
OS days (median)	1202	1188	1197	1427	1284

TCGA, The Cancer Genome Atlas; No. number; OS, overall survival; NA, not available.

### Development and verification of a prognostic risk score model

A total of 309 fatty acid–related genes were obtained from Molecular Signatures Database (MSigDB: http://www.gsea-msigdb.org/gsea/downloads.jsp). “Limma” under R condition was used to identify deferentially expressed genes (DEGs) between tumor and normal ovarian samples with adjusted *P* < 0.05 and |logFC|≥0.5. Then, the samples in TCGA dataset were randomly divided into training set (*n* = 228) and testing set (*n* = 151) according to the proportion of 6:4. After deleting the overlap patients and patients without survival information, there are 224 patients in training set and 144 patients in testing set. Training set was used to develop the prognostic risk score model. Prognostic-related DEGs of OS were selected by univariate Cox regression analysis (prognostic DEGs). LASSO (least absolute shrinkage and selection operator) Cox regression analysis was used to identify the independent prognostic factors with *P* < 0.05. Finally, backward stepwise selection with the Akaike information criterion (AIC) was used to reduce candidate genes (candidate DEGs) and constructed a multi-variable Cox regression model. The risk score (RS) can be calculated as follows: RS = $\sum_{\text{i}=1}^{\text{k}}{\text{β}}_{\text{i}}{\text{Exp}}_{\text{i}}$ , (Expi represents the expression level of each candidate genes and βi represents the corresponding regression coefficient). All cases in training set were divided into high- and low-risk groups with the median value of RS. Kaplan–Meier (K-M) survival curve and receiver operating characteristic (ROC) curve were used to identify the prognostic prediction value of the model. The association of the clinical information with RS was investigated in low- and high-risk groups. In addition, multivariable Cox regression analysis was used to identify that the risk score is an independent prognostic factor. The testing group and GSE26712 and GSE63885 were used for validation.

### Principal components analysis comparison

Firstly, “limma” package under R condition was used to perform principal components analysis (PCA) with the DEGs in training set and candidate DEGs in the fatty acid–related prognostic model. Then, “ggplot2” package was used to show the two-dimensional distinguishing capability of DEGs and candidate DEGs. The “ropls” package was used to calculate the *R* ^2^ and *Q* ^2^ values of PCA.

### Gene set variation analysis

Fatty acid metabolism-related gene sets (c2.cp.kegg.v7.1.symbols) from MSigDB (https://www.gsea-msigdb.org/gsea/msigdb) were downloaded as the reference genes. Gene set variation analysis (GSVA) was performed with “GSVA” package between low- and high-risk groups. *p* < 0.05 was regarded as statistically significant.

### Immune cell infiltrates and immune analysis

The RNA-seq data of ovarian cancer cases in TCGA database were uploaded to Timer2.0 (https://cistrome.shinyapps.io/timer/) to obtain the immune cells infiltration. Meanwhile, we applied “immunedeconv” package to conduct MCP-COUNTER algorithm (13) to get the abundance of both immune and stromal cells of each sample. The immune cells infiltration between low- and high-risk groups was compared. Then, ssGSEA (single sample gene set enrichment analysis) was performed using “GSVA” package to calculate enrichment scores that represent immune gene-related function in samples. We compared the enrichment scores between low- and high-risk groups.

### Association with treatment and immunotherapy response

“pRRophetic” package was used to calculate the halfmaximal inhibitory concentration (IC50), which can predict drug treatment response including the chemotherapy and targeting therapy. To analyze the association with immunotherapy response in high- and low-risk groups, first, we compared the expression of 12 common immune checkpoints (CTLA4, PDCD1, LAG3, TGFB1, IL10, TNFRSF14, IL13, CD244, CD48, ICAM1, NOS3, and MICB) (14) between the two groups. Then, the TIDE (Tumor Immune Dysfunction and Exclusion) value was assessed to show the immune escape of cancer cells and their response to immune checkpoint inhibitors (ICIs). RNA-seq data of ovarian cancer cases in TCGA database were uploaded to TIDE website (http://tide.dfci.harvard.edu/) to calculate TIDE value. Finally, IMvigor210 cohort was divided into low- and high-risk groups by the median of risk score, and immunity therapy effect was compared between groups.

### Development of prognostic-prediction nomogram

Nomogram was built with “rms” package to predict OS with RS and clinical factors including age, grade, stage, and debulking status. Calibration curve was shown to analyze the prediction accuracy. Multivariable Cox regression analysis was performed, and AUC of ROC curve was explored to identify the prognostic prediction value of nomogram.

### Enrichment analysis of the DEGs between the low- and high-risk groups

Differentially expressed genes between low- and high-risk groups were selected by “limma” package with *p* < 0.05. Gene ontology (GO) and Kyoto Encyclopedia of Genes and Genomes (KEGG) enrichment analysis were performed with “cluster- Profiler” package under R condition. The result was shown in barplot.

### RNA extraction and real-time quantitative polymerase chain reaction

Ovarian cancer cells including OVCAR3, SKOV3, and A2780 cells were cultured in DMEM (Gibco, C11995500BT, USA) supplemented with 10% fetal bovine serum (FBS) and 1% penicillin/streptomycin. Normal ovarian cell IOSE80 was maintained in RPMI 1640 (Gibco, C11875500BT, USA) supplemented with 10% FBS and 1% penicillin/streptomycin. Total RNAs were extracted from three cultured ovarian cancer cells and one normal ovarian cell type using the EZ-press RNA Purification Kit (EZBioscience, USA). cDNA synthesized the 4× reverse transcription master mix (EZBioscience, USA) in a 20-μl reaction system containing 1 μg of total RNA. Real-time quantitative polymerase chain reaction (RT-qPCR) was performed using the 2× SYBR Green qPCR Master Mix (EZBioscience, USA) in a 10-μl reaction system containing 1 μl of cDNA on a QuantStudio 5 RT-PCR System (Thermo Fisher Scientific, USA). RT-qPCR was performed by initial denaturation (5 min, 95°C), and 40 amplification cycles (10 s at 95°C and 30 s at 60°C). Melting curve analysis was used to verify the primer specificity. The threshold cycle (Ct) values of each cell type were used for the post-PCR data analysis. Relative gene(s) expression was identified and normalized against β-tubulin as the housekeeping gene. Real-time PCR primers are listed in **Table S1**.

### Statistical analysis

Wilcox Test was used to compare the continuous numerical data such as the expression level of mRNA, immune cells infiltration scores, risk scores between different groups. Chi-square test was used to compare discrete clinical parameters. *P*-values < 0.05 were considered statistically significant if not specified.

## Result

### Development of fatty acid metabolism-related prognostic signature

**Figure 1:**
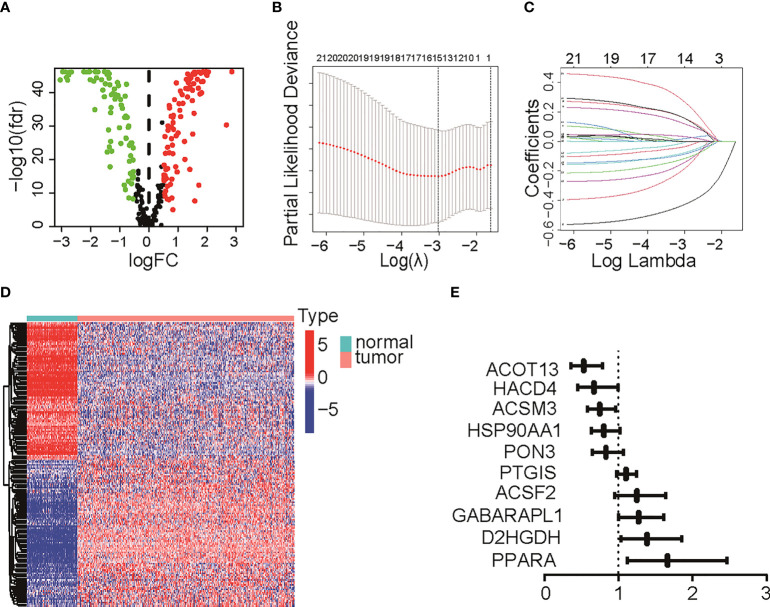
Differentially expressed genes (DEGs) identification and Lasso Cox regression. **(A)** Volcano plot for DEGs between normal tissue and tumor. Red pot represents DEGs with adjusted *P* < 0.05 and |logFC|≥0.5. **(B, C)** LASSO Cox regression to select independent prognostic-related genes. **(D)** Heat map of DEGs with adjusted *P* < 0.05 and |logFC|≥0.5. The color red represents high-expression genes and color green represents low-expression genes. **(E)** HR of 10 final candidate genes involved in survival model. LASSO, least absolute shrinkage and selection operator; DEG, differentially expression genes; FC, fold change; HR, hazard ratio.

**Figure 2:**
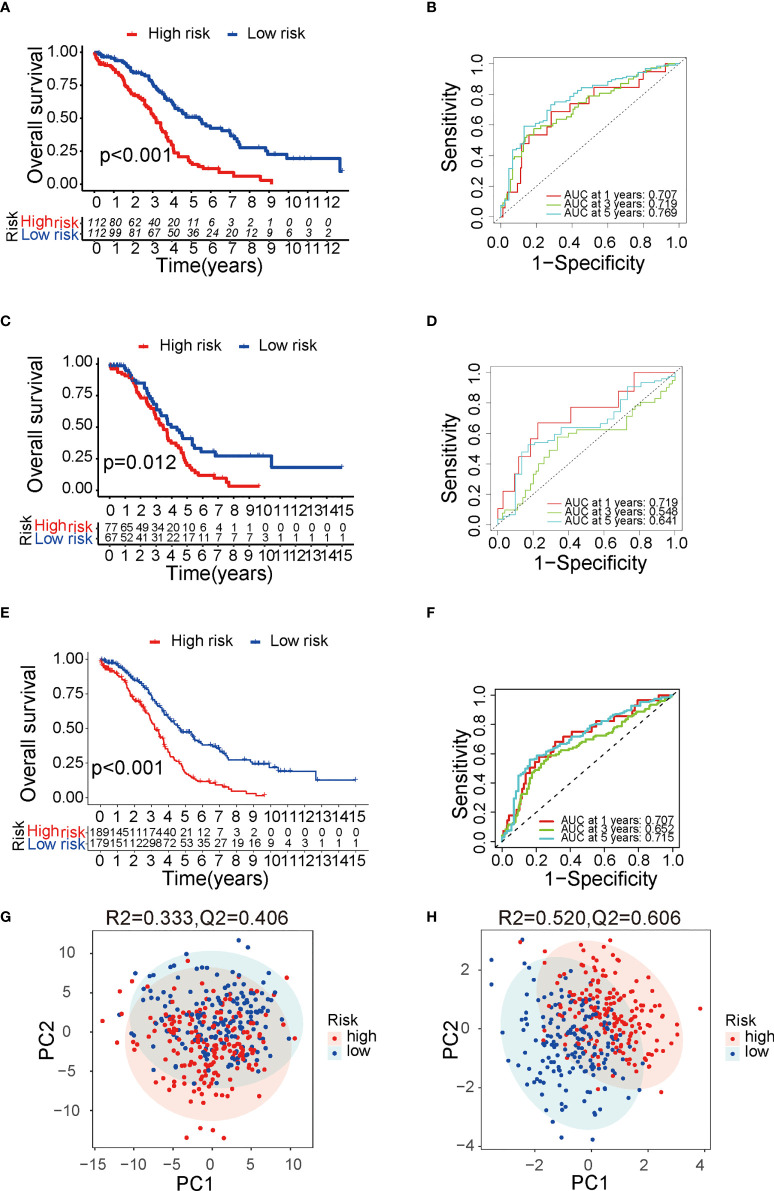
Verification of the prognostic risk score model. **(A)** The Kaplan–Meier survival curve of TCGA training group. **(B)** The ROC curve of TCGA training group. **(C)** The Kaplan–Meier survival curve of TCGA testing group. **(D)** The ROC curve of TCGA testing group. **(E)** The Kaplan–Meier survival curve of TCGA cohort. **(F)** The ROC curve of TCGA cohort. **(G)** PCA result with all DEGs with 95% CI ellipses. **(H)** PCA result with the final candidate genes of the model with 95% CI ellipses. TCGA, The Cancer Genome Atlas; ROC, receiver operating characteristic; DEG, differentially expressed genes; PCA, principal components analysis.

The TME is characterized by alteration of fatty acid metabolism. To illustrate the prognostic role of fatty acid metabolism, we tested the expression of fatty acid metabolism-related genes between 379 ovarian cancer samples from TCGA dataset and 88 normal samples from GTEx dataset (details shown in **Table 1**). We identified 176 differentially expressed genes (DEGs), including 85 downregulated and 91 upregulated genes (**Figures 1A, D** and **Table S2**). Then, we performed univariate Cox analysis in TCGA training set and identified a total of 22 prognostic-DEGs related to OS selected with *p* < 0.1 among 176 DEGs (**Figure S1A**). Furthermore, 16 independent prognostic-related factors were addressed with LASSO Cox regression analysis (**Figures 1B, C**). Backward stepwise selection with the AIC was then applied, and a multi-variable Cox regression model was constructed with 10 independent candidate genes, including HACD4, PON3, ACSF2, ACOT13, GABARAPL1, ACSM3, D2HGDH, PTGIS, PPARA, and HSP90AA1 (**Figure 1E**). The corresponding coefficients were shown in **Table S3**, and the risk score of each sample was calculated: risk-score = (-0.40) × exp(HACD4) + (-0.18) × exp(PON3) + 0.22 × exp(ACSF2) + (-0.63) × exp(ACOT13) + 0.24 × exp(GABARAPL1) + (-0.29) × exp(ACSM3) + 0.33 × exp(D2HGDH) + 0.10 × exp(PTGIS) + 0.51 × exp(PPARA) + (-0.22) × exp(HSP90AA1). Thus. all cancer samples in training set were divided into two groups (low- and high-risk groups) by the median value of risk score. Although there were no statistically significant differences between low- and high-risk groups associated with age, stage, grade, and debulking status (**Figure S1B**), high-risk group had a poor prognosis when comparing with low-risk group as shown with K-M survival curve (*p* < 0.01) (**Figure 2A**). This prognostic signature showed good prediction value with AUC > 0.7 in ROC curve (**Figure 2B**). Furthermore, the testing set from TCGA was used to validate the model, illustrating the good prediction value of this fatty acid metabolism-related prognostic signature in ovarian cancer (**Figures 2C, D**), as also shown with the whole TCGA cohort (**Figures 2E, F**) and independent GEO datasets (**Figure S1I**). We also analyzed the predictive power of top 5 genes that contributed most to the model (with the maximum absolute value of coefficient) through AUC of ROC curves (**Figure S1J**). The 10 candidate genes in the prognostic model (*R* ^2^ = 520, *Q* ^2^ = 0.606) showed better distinguishing capability than all DEGs (*R* ^2^ = 0.333, *Q* ^2^ = 0.406) as shown in PCA results (**Figures 2G, H**). Uni- and multi-variable Cox regression analysis conducted on training set, testing set and the whole TCGA cohort addressed that the risk score is an independent prognostic factor (**Figures S1C–H**) after adjusting for clinical characters. Remarkably, we constructed a metabolic map summarizing the fatty acid metabolism (**Figure S2A**), and most of the independent candidate genes were marked in the position where they are functioning in the fatty acid metabolism process.

### Development of a nomogram to predict OS

**Figure 3:**
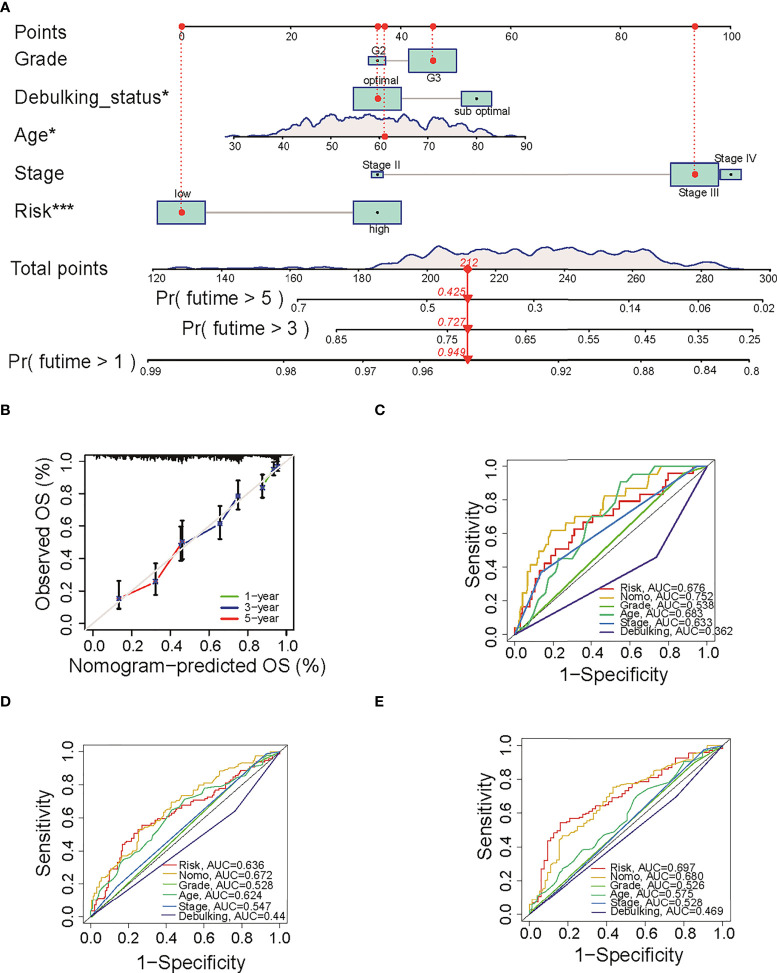
The predictive value of nomogram in OS. **(A)** The constructed prognostic nomogram that predicts 1-, 3-, and 5-year OS of ovarian cancer patients in TCGA cohort. **(B)** The calibration plots of the nomogram. The *x*- and *y*-axis present predicted and actual survival respectively. **(C–E)** ROC curve of 1-, 3-, and 5-year OS with the nomogram, the clinical characters, and risk score. OS, overall survival; TCGA, The Cancer Genome Atlas; ROC, receiver operating characteristic. *P < 0.05 and ***P < 0.001.

To predict the OS for ovarian cancer accurately, a prognostic nomogram was constructed based on the parameters including grade, stage, age, sub-optimal debulking status, and risk score (**Figure 3A**). This nomogram model showed a good prediction value, as shown in the calibration curve for 1-, 3- and 5-year OS (**Figure 3B**). The AUC in ROC curve of the nomogram is 0.752 for 1-year, 0.672 for 3-year, and 0.680 for 5-year OS, which was larger than that of single clinical character or risk score (0.676 for 1 year, 0.636 for 3 years, and 0.697 for 5 years, separately) (**Figures 3C–E**), indicating that the nomogram model has a better prediction value than clinical characters or risk score alone. In addition, uni- (**Figure S2B**) and multi-variable Cox regression analysis (**Figure S2C**) suggested that nomogram is an independent prognostic factor adjusting for other clinical characters.

### Immune infiltration between low- and high-risk groups

**Figure 4:**
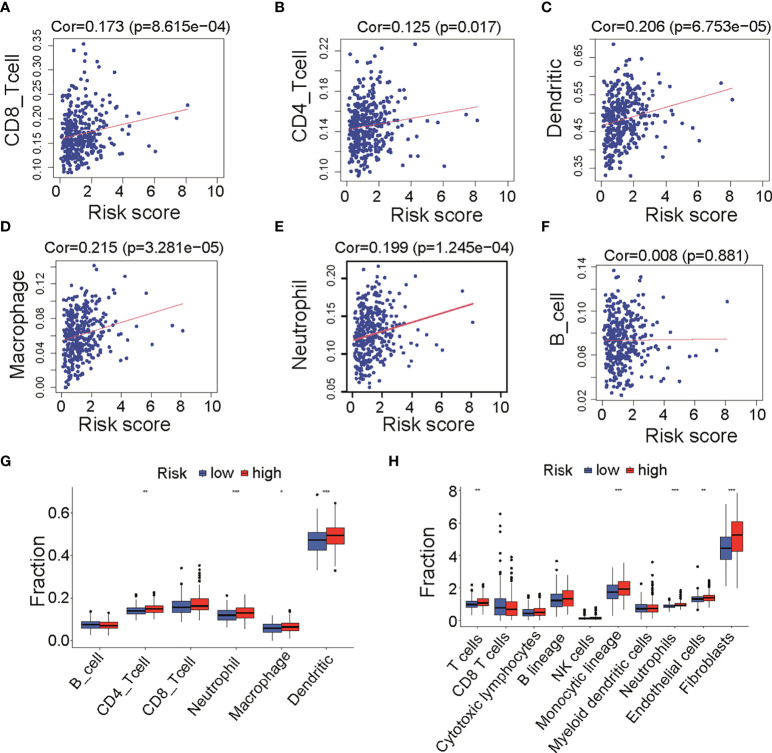
Infiltration of immune cells between low- and high-risk groups in TCGA cohort. **(A–F)** Correlation analysis of risk score and six immune cells infiltration from TIMER, including CD8 T cells **(A)**, CD4 T cells **(B)**, dendritic cells **(C)**, macrophages **(D)**, neutrophil **(E)**, and B cells **(F)**. **(G)** Differences of six immune cells infiltration (TIMER) among low- and high-risk groups. **(H)** Differences of immune cells and stromal cells infiltration (MCP-COUNTER) among low- and high-risk groups. TCGA, The Cancer Genome Atlas; TIMER, Tumor Immune Estimation Resource; MCP-COUNTER, Microenvironment Cell Populations-counter. *P < 0.05, **P < 0.01 and ***P < 0.001.

As shown in **Figures 2A, C, E**, cases in high-risk group had poor prognosis but had no relationship with clinical factors such as grade, stage, and debulking status (**Figure S1B**). We wonder if fatty acid metabolism affects the cancer prognosis *via* influencing tumor micro-environment in ovarian cancer. Published studies showed that anti-tumor antigen vaccination targeting immune cells constitutes an efficient therapy strategy for malignant cancer. To prove the critical role of immune cell infiltration in micro-environment, we analyzed the infiltration of six immune cells including B cells, CD4 T cells, CD8 T cells, neutrophils, macrophages, and dendritic cells that were obtained from the Timer 2.0 database. Results showed that CD8 T cells (Cor = 0.178, *p* = 8.615e-4, **Figure 4A**), CD4 T cells (Cor = 0.125, *p* = 0.017, **Figure 4B**), dendritic cells (Cor = 0.206, *p* = 6.753e-5, **Figure 4C**), macrophages (Cor = 0.215, *p* = 3.28e-5, **Figure 4D**), and neutrophils (Cor = 0.199, *p* = 1.245e-4, **Figure 4E**) were positively correlated with risk score, whereas B cells were not correlated (**Figure 4F**). The result of immune cells enrichment between two groups (**Figure 4G**) was similar to those of correlation analysis (**Figures 4A–E**). The enrichment of CD4 T cells, neutrophils, macrophages, and dendritic cells in high-risk group was likely due to a much higher immune-active status. In addition, to validate the abundance of immune cells in two groups, we compared 10 kinds of cells including immune and stromal cells from MCP-COUNTER. Results showed that T cells, monocytic lineage, neutrophils, endothelial cells, and fibroblasts were enriched in high-risk group (**Figure 4H**), consistent with results above (**Figure 4G**). Remarkably, T cells (Cor = 0.146, *p* = 0.005), Monocytic lineage (Cor = 0.233, *p* = 6.283e-06), neutrophils (Cor = 0.264, *p* = 2.673e-07), endothelial cells (Cor = 0.198, *p* = 1.347e-04), and fibroblasts (Cor = 0.342, *p* = 1.474e-11) were positively correlated with risk score (**Figure S3**), consistent with results from Timer 2.0. Furthermore, as immune function has been implicated to play a pivotal role in T-cell dysfunction and T-cell signaling (15), we assessed the role of immune function between two groups. Results showed that APC co-stimulation, T cell co-stimulation, and Type II IFN Response were more enriched in high-risk group (**Figure S4**). Collectively, these results suggested that different immune cells regulated immune function in two groups with different fatty acid metabolism status, which appears to be an important component of tumor micro-environment.

### Immunotherapy analysis between low- and high-risk groups in TCGA cohort

**Figure 5:**
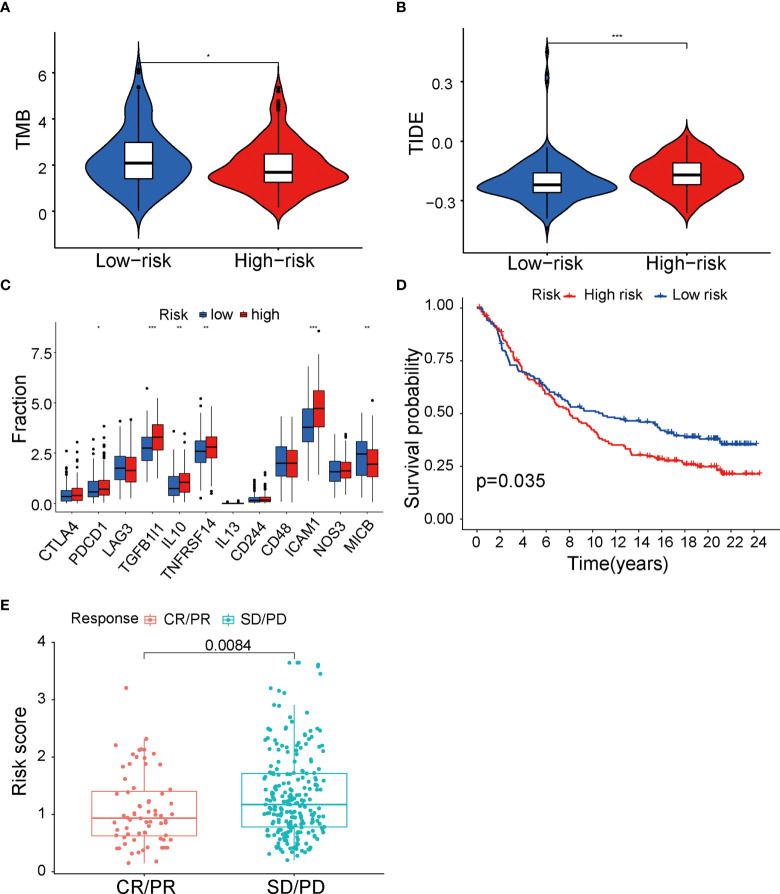
Immunity therapy analysis between low- and high-risk groups in TCGA cohort. **(A)** Differences of 12 immune checkpoints among low- and high-risk groups. **(B)** TMB value between low- and high-risk groups. **(C)** TIDE value between low- and high-risk groups. **(D)** The Kaplan–Meier survival curve of low- and high-risk group in IMvigor210 cohort. **(E)** Differences of risk score among CR+PR and SD+PD groups. TCGA, The Cancer Genome Atlas; TMB, tumor mutational burden; TIDE, Tumor Immune Dysfunction and Exclusion; CR, complete response; PR, partial response; SD, stable disease; PD, progression disease. *P < 0.05, **P < 0.01 and ***P < 0.001.

It is well established that different immune cells infiltration promotes immune function alteration, leading to significant change of immunotherapy response. To provide evidence supporting the role of fatty acid metabolism on immunotherapy response, first, we performed analysis on immune checkpoints expression between two groups. As indicated, ICIs against PD-1, PD-L1, CTLA-4, and Lag3 have becoming the promising strategy for the treatments of a variety of malignancies. Cancer cells can activate immune checkpoint pathways and induce immunosuppressive functions, thus targeting immune checkpoint pathways provides a promising therapeutic breakthrough in cancer. Indeed, the expression of immune checkpoints may be related to the therapy response. We analyzed the expression of 12 common immune checkpoints between two groups. As seen in **Figure 5A**, PDCD1, TGFB1I1, IL10, TNFRSF14, and ICAM1 were significantly higher expressed in high-risk group, whereas MICB was significantly lower expressed, indicating the heterogeneity of immune status and different therapy response correlating with different immune checkpoints expression (**Figure 5A**). Next, we tested whether TMB (tumor mutation burden) changed between two groups. TMB is known to be positively correlated with immunotherapy sensitivity and efficacy in cancer. Thus, higher TMB in low-risk group indicated that cases in this group had good immunotherapy efficacy (**Figure 5B**). Additionally, we performed analysis with TIDE value to access the immunotherapy efficacy, especially the well-known PD-1 and CTLA-4 inhibitors. TIDE value is implicated to be positively related with the ability of immune escape of cancer cells. Results showed that high-risk group had higher TIDE value (**Figure 5C**), indicating that immune cells in high-risk group may have the potential of immune escape and be resistant to immunotherapy. Furthermore, to directly explore the potential association between ICIs’ sensitivity and risk score in the signature, patients who received anti–PD-L1 immune therapy in the IMvigor210 cohort were divided into high- and low-risk groups by the median of risk score in the model. K-M survival curve was performed, and the result showed that high-risk group had poor prognosis (*p* = 0.035, **Figure 5D**), consistent with our analysis above that risk score in our fatty-acid signature is related with the sensitivity of ICIs’ sensitivity. Meanwhile, results with box plot corroborated our K-M curve findings that the objective response group, which included partial response (PR) and complete response (CR) to ICIs, had lower risk score than stable disease/progression disease group (*p* = 0.0084) (**Figure 5E**). Taken together, our results strongly supported the role of risk score of the fatty acid–related signature in identification of cases with immunotherapy response.

### Drug sensitivity analysis between low- and high-risk groups

**Figure 6:**
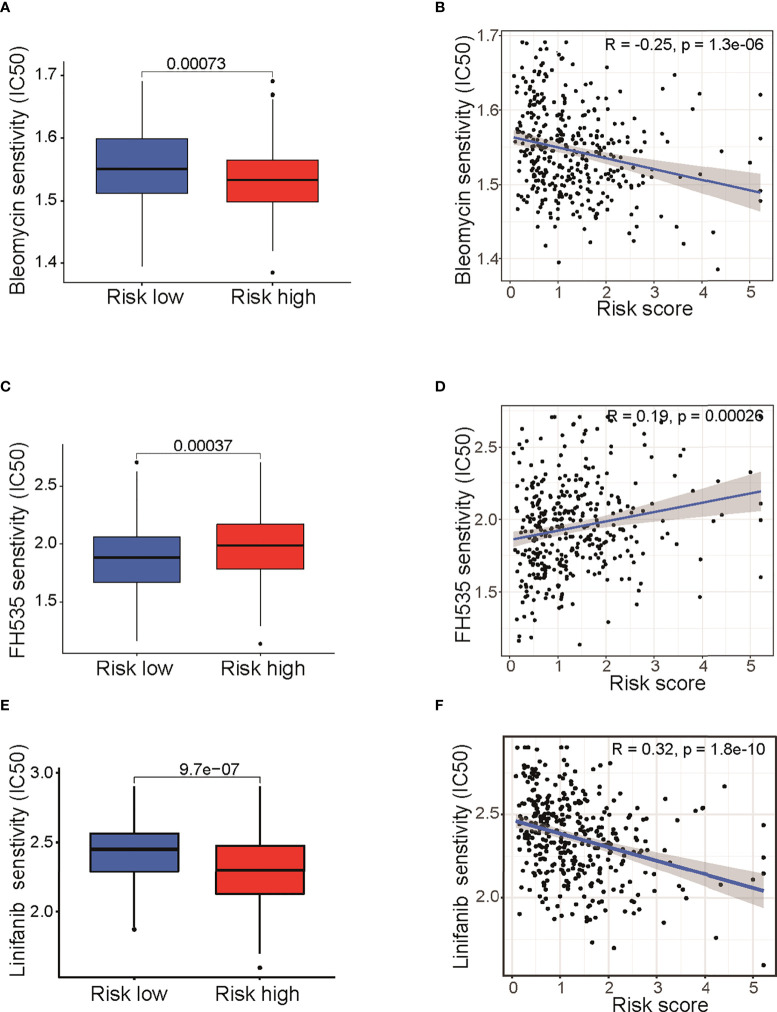
Drug sensitivity analysis between low- and high-risk groups. Differences of IC50 for Bleomycin **(A)**, FH535 **(C)**, and Linifanib **(E)**.Correlation analysis of risk score and IC50 of Bleomycin **(B)**, FH535 **(D)**, and Linifanib **(F)**. IC50: half-maximal inhibitory concentration; FH535 (PPAR inhibitor); Linifanib (VEGFR inhibitor).

It is well known that the standard treatment of patients with epithelial ovarian cancer comprises debulking surgery followed by chemotherapy and maintained by targeted therapy. To begin to elucidate the prediction value of fatty acid–related genes for chemotherapy or targeted therapy response, we calculated the half maximal inhibitory concentration (IC50) with “pRRophetic” package under R condition. Results indicated that risk score in fatty acid gene-related model was correlated with treatment response of multiple chemotherapy or targeted therapy reagents (**Table S4**). Remarkably, bleomycin, which showed good efficacy in ovarian cancer (16), had different IC50 between two groups (*P* < 0.001) (**Figure 6A**) and was negatively correlated with risk score (**Figure 6B**). In addition, targeted therapies, such as FH535 (PPAR inhibitor) (**Figures 4C, D**) and Linifanib (VEGFR inhibitor) (**Figures 4E, F**), had different therapy efficacy and were correlated with risk score. Collectively, these results indicated that risk score in the prognosis model is related with therapy efficacy, thus affecting the therapy choice. Furthermore, biological pathways including MAPK signaling pathway, VEGF signaling pathway, and mTOR signaling pathway were enriched in high-risk group (**Figure S5**). The enrichment of biological pathways was consistent with the drug sensitivity results (**Table S4** and **Figure 6**), showing that TAK-715 (MAPK inhibitor) and XL-184 (VEGF inhibitor) had good efficacy on cancer treatment.

### Enrichment analysis of the DEGs between the low- and high-risk groups

To elucidate the mechanism of fatty acid gene-mediated regulation, enrichment analysis with the DEGs between the low- and high-risk groups was performed. We identified 465 DEGs between low- and high-risk groups with adjusted *P* < 0.05 and |logFC| ≥ 0.5, including 109 downregulated and 354 upregulated genes (**Figure S6**). The GO analysis and KEGG analysis were conducted. Results showed that numerous DEGs were involved in extracellular matrix organization, ECM−receptor interaction, PI3K−Akt signaling pathway and TNF signaling pathway (**Figure S7**).

### Candidate genes expression are aberrantly altered in ovarian cancer cells

**Figure 7:**
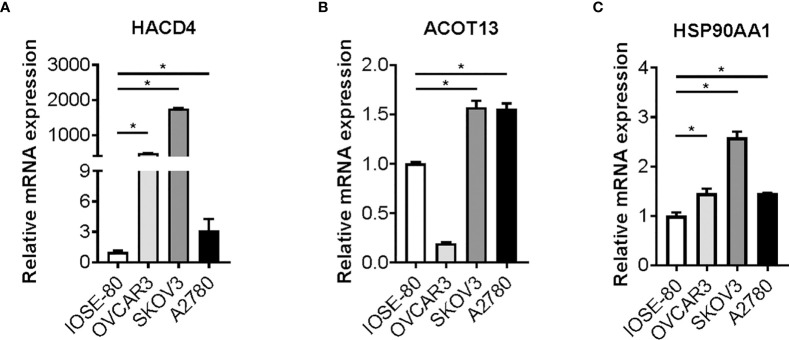
Candidate genes expression in ovarian cancer cells. Gene expression of HACD4 **(A)**, ACOT13 **(B)**, and HSP90AA1 **(C)** in ovarian cancer cells (OVCAR3, SKOV3, and A2780 cells) *versus* normal ovarian cells (IOSE80 cells) determined by RT-qPCR analysis. RT-qPCR, reverse transcription and quantitative real-time polymerase chain reaction. *P < 0.05.

To validate the characteristic and function of fatty acid in ovarian cancer cells, we tested whether the expression of ten candidate genes is altered in cancer cells. We cultured and maintained ovarian cancer cells (OVCAR3, SKOV3 and A2780 cells) and normal ovarian cells (IOSE 80), and RNAs were extracted from the cells above. Expression of candidate genes was determined by reverse transcription and RT-qPCR analysis. Strikingly, a statistically significant increase in SKOV3 and A2780 cells *versus* matched IOSE80 cells was observed in the three bio-informatively proved upregulated genes including HACD4, ACOT13, and HSP90AA1 (**Figure 7**). Meanwhile, most of bio-informatively proved downregulated genes were statistically significant decreased in OVCAR3 and A2780 cells *versus* matched IOSE80 cells, suggesting the critical role of these candidate genes in ovarian cancer (**Figure S8**).

## Discussion

Fatty acid metabolism alterations in cancer cells have been increasingly being recognized. Emerging evidence suggests that fatty acid metabolism which includes fatty acid synthesis, degradation, and uptake process, provides energy storage, membrane proliferation and signaling transduction for cancer and immune cells and underlies the pathogenesis and development of ovarian cancer (17). Fatty acid synthesis and uptake pathways may be the potential targets for cancer therapy strategies. In the recent years, studies have been conducted to explore the role of fatty acid metabolism in ovarian cancer. For example, fatty acid binding protein 4 (FABP4), a lipid chaperone protein, has been regarded as a critical regulator to adapt and colonize TME and is implicated and applied in ovarian cancer for the providence of fatty acids (FAs) from surrounding adipocytes to tumor cells (18). Targeting FABP4 can restrict ovarian cancer metastasis as a specific metabolic target (19). The tumor-progression process in cancer may involve the interplay between multiple cells, factors, and the TME. However, published studies were focused on the specific factor or gene. Thus, the integrated analysis of fatty acid metabolism is needed. Remarkably, we systematically analyzed the fatty acid metabolism-related genes in ovarian cancer and found that they were differently expressed. Therefore, the prognostic-related model with risk score was defined according to the candidate fatty acid metabolism-related DEGs including HACD4, PON3, ACSF2, ACOT13, GABARAPL1, ACSM3, D2HGDH, PTGIS, PPARA, and HSP90AA1. This prognostic-related model and the constructed nomogram can predict ovarian cancer prognosis and therapy response including chemotherapy, targeted therapy, and immunotherapy. Furthermore, we validated the expression of 10 candidate genes in ovarian cancer cells compared with normal ovarian cells. Together, these findings strongly point to a critical role of fatty acid metabolism in ovarian cancer prognosis and therapy response.

The way to limit FA availability included decreasing FA synthesis, inducing FA degradation by oxidation, increasing FA to storage, and blocking FA release from storage. The genes and enzymes in FA availability process consist of ATP citrate lyase, acetyl-CoA carboxylase, acyl-CoA synthetase, fatty acid synthase, and transcription factors for FA synthesis (20). The candidate DEGs included in our model were included in this process. Among them, ACSF2 and ACSM3 are Acyl-CoA Synthetase Family Member and involved in fatty acid biosynthesis process. PON3 is applied for arachidonic acid metabolism. D2HGDH can be applied for inter-conversion of 2-oxoglutarate and 2-hydroxyglutarate and included in pyruvate metabolism and citric acid (TCA) cycle, rendering the fatty acid synthesis. ACOT13, HACD4, and PPARA are considered to be involved in the process of FA degradation, inducing FA oxidation as major transcriptional regulators (21, 22). GABARAPL1 enables ubiquitin protein ligase binding, promoting lipolysis of FA to serve as precursors for important signaling lipids (23). Thus, the fatty acid metabolism gene-related model was constructed and may be the representative one reflecting the fatty acid metabolism reprogramming in ovarian cancer. This model divided ovarian cancer cases into high- and low-risk groups that had different prognostic status. Furthermore, the testing set in TCGA dataset validated the model intrinsically, whereas the GEO datasets validated this extrinsically. In addition, this prognostic model is the independent prognostic factor, reflecting that fatty acid metabolism has crucial value in ovarian cancer progression and pathogenesis. The more accurate nomogram may be used in clinical for predicting the prognosis of specific ovarian cancer case, thus rendering the new choice for therapy strategies.

Ovarian cancer has the features of high chemotherapy resistant and relapse rate. Recently, more studies explored that fatty acid metabolism in TME and lipid composition of cellular membranes was linked to chemotherapeutic agents’ response and resistance, but clinical data linking fatty acid metabolism to therapy resistance in tumors remained elusive. Based on the available data in previous study on single specific gene, fatty acid metabolism reprogramming has drawn significant attention as essential mediators of chemoresistant cancer cases. Our recent studies showed that model constructed based on fatty acid metabolism-related genes linked to response of chemotherapy agents. In addition, our study demonstrated that the fatty acid metabolic characteristics of ovarian cancer presented considerable hurdles to immune cells infiltration mainly including macrophages, T cells, neutrophils, and monocytic lineage. Macrophages, the infiltrates in high-risk group, are proved to play pivotal roles in inflammatory processes. As an independent molecule in the anti-inflammatory fatty acid biosynthesis, SREBP1 contributes to the resolution of TLR4-induced gene activation by macrophage fatty acid metabolism reprogramming (24). By contrast, glutamine blocking in tumors linked to oxidative metabolism and exposed an undefined metabolic plasticity between effector T cells and cancer cells (25). Immune cells differed between two groups, thereby regulating immune response and contributing to immunotherapy strategies. Thus, fatty acid metabolism antagonism may be exploited as the “metabolic checkpoint” for tumor therapy. Here, we further explored the correlation of fatty acid metabolism and immune checkpoint inhibitors response. Results showed that high-risk group had lower TMB, higher TIDE, and different expression of checkpoint genes, reflecting that cancer cells in high-risk group had the characteristics of immune evasion and lower immunotherapy response, consistent with the result from exploration of ICIs response. The exploration of fatty acid metabolism patterns in ovarian cancer and the role of fatty acid metabolism in cancer cell immunity could help to understand the mechanism of fatty acid metabolism in OC progression, thus guiding to an effective therapeutic strategy. Moreover, due to the heterogeneity within individual patient of immunotherapy response, attempts should be made to select the cases with good efficacy. Thus, our study highlighted the role of metabolic alteration in ovarian cancer pathogenesis and presented a fatty acid gene-related model with good prediction value for immunotherapy efficacy.

To explore the biological process under fatty acid metabolic reprogramming, GO and KEGG pathway analyses of the DEGs between these two groups showed that extracelluar matrix (ECM)–receptor interaction, focal adhesion, and engulfment phagocytosis is significantly enriched. Publications indicate that ECM homeostasis is maintained by the complex integration of cytokine and environmental mediators including fatty acid oxidation. FAO pathway activation generates an inhibition of ECM transcription and induced ECM internalization and degradation (26). Our findings are highly consistent with publications about the metabolic perturbation of ECM homeostasis. Additionally, KEGG and GSVA analyses demonstrated that the main pathways affected by fatty acid metabolism are PI3K/AKT/mTOR and AMPK pathways. Oncogenic activation of PI3K-AKT-mTOR signaling suppresses oxidative stress of cancer cells through lipogenesis, showing therapeutic promise in cancer (27). Interestingly, dysregulated PI3K-Akt-mTOR signaling in cancer has been increasingly utilized for developing targeted therapies (28). Inhibitors targeting this signaling demonstrated the role for targeting T-cell immune signaling and attenuating immune cell effector function (29). Thus, it is helpful to explore the mechanism underlying the fatty acid metabolism reprogramming in tumor environment in clinical therapy.

Although we found the close association between fatty acid metabolism and cancer cell immunity, further specific mechanisms under fatty acid reprogramming were not explored. Meanwhile, our study is based on the integrative analysis of publicly datasets with large sample sizes, a specificmodel with interventions on fatty acid metabolism may be constructed to study the influence of metabolic reprogramming on disease and cancer cell immunity.

## Conclusion and perspectives

Fatty acid metabolism has been increasing appreciated for the profoundly influences for tumor progression and metastasis *via* oxidation and fatty acids synthesis. Specifically, fatty acid metabolism reprogramming is implicated in cancer cell immunity and immune cells infiltration in TME. Importantly, changes in fatty acid metabolism patterns are indicated in treatment resistance especially immunotherapy, thus targeting fatty acid metabolism may overcome therapy resistance and may be particularly a future approach for co-targeting strategies. Our integrative analysis focused on the fatty acid metabolism pattern in ovarian cancer and demonstrated the crucial interaction between cancer cells immunity and metabolism in TME, enhancing our understanding of fatty acid metabolism reprogramming in treatment response and resistance. Future investigations may explore to overcome the deficiencies with therapy resistance and off-target effects of current clinical inhibitors. Moreover, patient-based prediction model is necessary to be employed to identify the specific resistant or hyper-reactive cases. Finally, attempts linking the genetic profiling to specific molecular characteristics and subtypes may decrease the complex heterogeneity and render personalized prognostication and management.

## Data availability statement

The original contributions presented in the study are included in the article/**Supplementary Material**. Further inquiries can be directed to the corresponding author.

## Author contributions

TC and JD designed the experiments and wrote the paper. TC, JD, JH and ZT performed the experiments, collected the data, analyzed data and prepared tables and figures. HS critically reviewed and revised the manuscript. All authors contributed to the article and approved the submitted version.

## Acknowledgments

The authors acknowledge the contribution of all databases including TCGA, GTEx, and GEO that provide free online tools and resources. This work was supported by GuangDong Basic and Applied Basic Research Foundation 2019A1515110312 [TC] and National Natural Science Foundation of China 82002734 [TC].

## Conflict of interest

The authors declare that the research was conducted in the absence of any commercial or financial relationships that could be construed as a potential conflict of interest.

## Publisher’s note

All claims expressed in this article are solely those of the authors and do not necessarily represent those of their affiliated organizations, or those of the publisher, the editors and the reviewers. Any product that may be evaluated in this article, or claim that may be made by its manufacturer, is not guaranteed or endorsed by the publisher.

## Supplementary material

PCR data-Relative gene expression https://www.jianguoyun.com/p/DfeK9VYQvdHdChjX-8oEIAA

Supplementary Figure 1 Prognostic-related DEGs in TCGA training group and uni-variable and multi-variable Cox regression survival analysis. **(A)** Unadjusted HRs (boxes) and 95% CI (horizontal lines) limited to prognostic related DEGs from TCGA training group by using uni-variable COX analysis. **(B)** Heat map of the association between 10 candidate-gene expression and clinical characters among low- and high-risk groups. Uni-variable Cox regression analysis in TCGA training group **(C)**, TCGA testing group **(E)**, TCGA cohort **(G)**. Multi-variable Cox regression analysis in TCGA training group **(D)**, TCGA testing group **(F)**, TCGA cohort **(H)**. **(I)** The Kaplan–Meier survival curve of OS in the GEO validation set. **(J)** The ROC curve of TCGA training group with top 5/10 genes. TCGA: The Cancer Genome Atlas; DEG: differentially expressed genes; ROC: receiver operating characteristic; GEO: Gene Expression Omnibus.

Supplementary Figure 2 The fatty acid metabolic map model and uni-variable and multi-variable Cox regression survival analysis of the nomogram. **(A)** The fatty acid metabolic map model and the candidate genes were marked in the position where they are functioning in the fatty acid metabolism process (pink box). Uni-variable Cox regression **(B)** and multi-variable Cox regression **(C)** analysis of nomogram with clinical characters in TCGA cohorts. TCGA: The Cancer Genome Atlas; TG: Triacylglycerols; TCA: Tricarboxylic acid cycle.

Supplementary Figure 3 Correlation analysis of risk score and infiltration of seven immune and stromal cells from MCP-COUNTER. Correlation analysis between B lineage **(A)**, endothelial cells **(B)**, fibroblasts **(C)**, monocytic lineage **(D)**, myeloid dendritic cells **(E)**, neutrophils **(F)**, and T cells **(G)**. MCP-COUNTER: Microenvironment Cell Populations-counter.

Supplementary Figure 4 Differences of immune-related functions among low- and high-risk groups in TCGA cohorts. TCGA: The Cancer Genome Atlas.

Supplementary Figure 5 Comparison of GSVA score between low-risk and high-risk groups. GSVA: gene set variation analysis.

Supplementary Figure 6 Differentially expressed genes (DEGs) among low- and high-risk groups in TCGA cohorts. **(A)** Volcano plot for DEGs. Red pot represents DEGs with adjusted *P* < 0.05 and |logFC|≥0.5. **(B)** Heat map of the DEGs among low- and high-risk groups. The color red represents high-expression genes and color green represents low-expression genes. TCGA: The Cancer Genome Atlas; DEGs: differential expression genes; FC: fold change.

Supplementary Figure 7 Enrichment analysis of the DEGs between the low- and high-risk groups. **(A)** Bar plot of GO analysis. **(B)** Bar plot of KEGG analysis. DEG: differentially expressed genes; GO: gene ontology; KEGG: Kyoto encyclopedia of genes and genomes.

Supplementary Figure 8 Candidate genes expression in ovarian cancer cells. Gene expression of PON3 **(A)**, ACSF2 **(B)**, ACSM3 **(C)**, D2HGDH **(D)**, GABARAPL1 **(E)**, PTGIS **(F)**, and PPARA **(G)** in ovarian cancer cells (OVCAR3, SKOV3, and A2780 cells) *versus* normal ovarian cells (IOSE80 cells) determined by RT-qPCR analysis. RT-qPCR: reverse transcription and quantitative real-time polymerase chain reaction.
