# Supplementary figures and images for: Identification of fatty acid signature to predict prognosis and guide clinical therapy in patients with ovarian cancer
Source: Front Oncol. 2022 Oct 4;12:979565. doi: 10.3389/fonc.2022.979565 (PMC9577003; doi:10.3389/fonc.2022.979565)

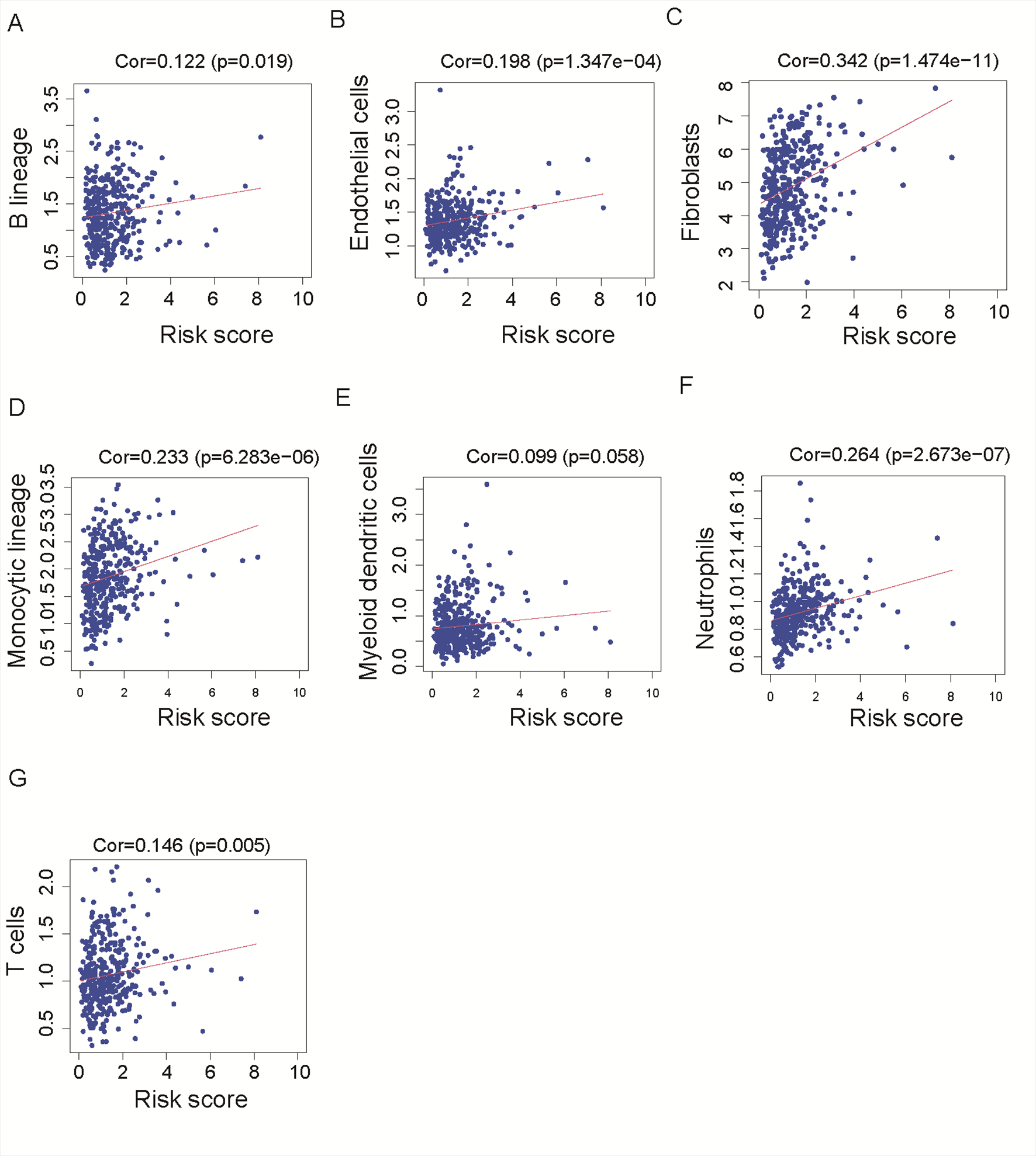

Supplement: Supplementary Figure 1 — Prognostic-related DEGs in TCGA training group and uni-variable and multi-variable Cox regression survival analysis. (A) Unadjusted HRs (boxes) and 95% CI (horizontal lines) limited to prognostic related DEGs from TCGA training group by using uni-variable COX analysis. (B) Heat map of the association between 10 candidate-gene expression and clinical characters among low- and high-risk groups. Uni-variable Cox regression analysis in TCGA training group (C), TCGA testing group (E), TCGA cohort (G). Multi-variable Cox regression analysis in TCGA training group (D), TCGA testing group (F), TCGA cohort (H). (I) The Kaplan–Meier survival curve of OS in the GEO validation set. (J) The ROC curve of TCGA training group with top 5/10 genes. TCGA: The Cancer Genome Atlas; DEG: differentially expressed genes; ROC: receiver operating characteristic; GEO: Gene Expression Omnibus. [file Image_1.tif]

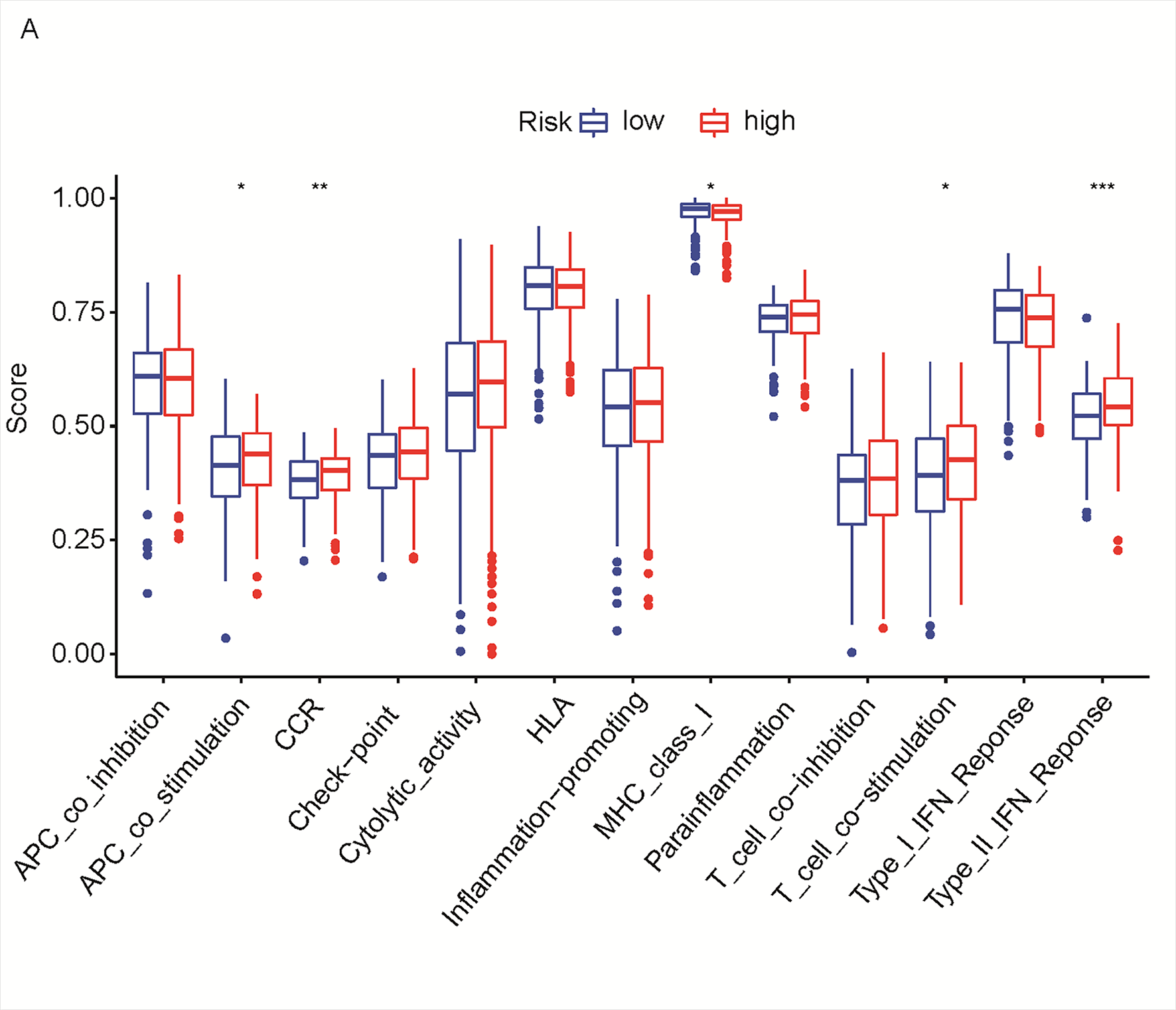

Supplement: Supplementary Figure 2 — The fatty acid metabolic map model and uni-variable and multi-variable Cox regression survival analysis of the nomogram. (A) The fatty acid metabolic map model and the candidate genes were marked in the position where they are functioning in the fatty acid metabolism process (pink box). Uni-variable Cox regression (B) and multi-variable Cox regression (C) analysis of nomogram with clinical characters in TCGA cohorts. TCGA: The Cancer Genome Atlas; TG: Triacylglycerols; TCA: Tricarboxylic acid cycle. [file Image_2.tif]

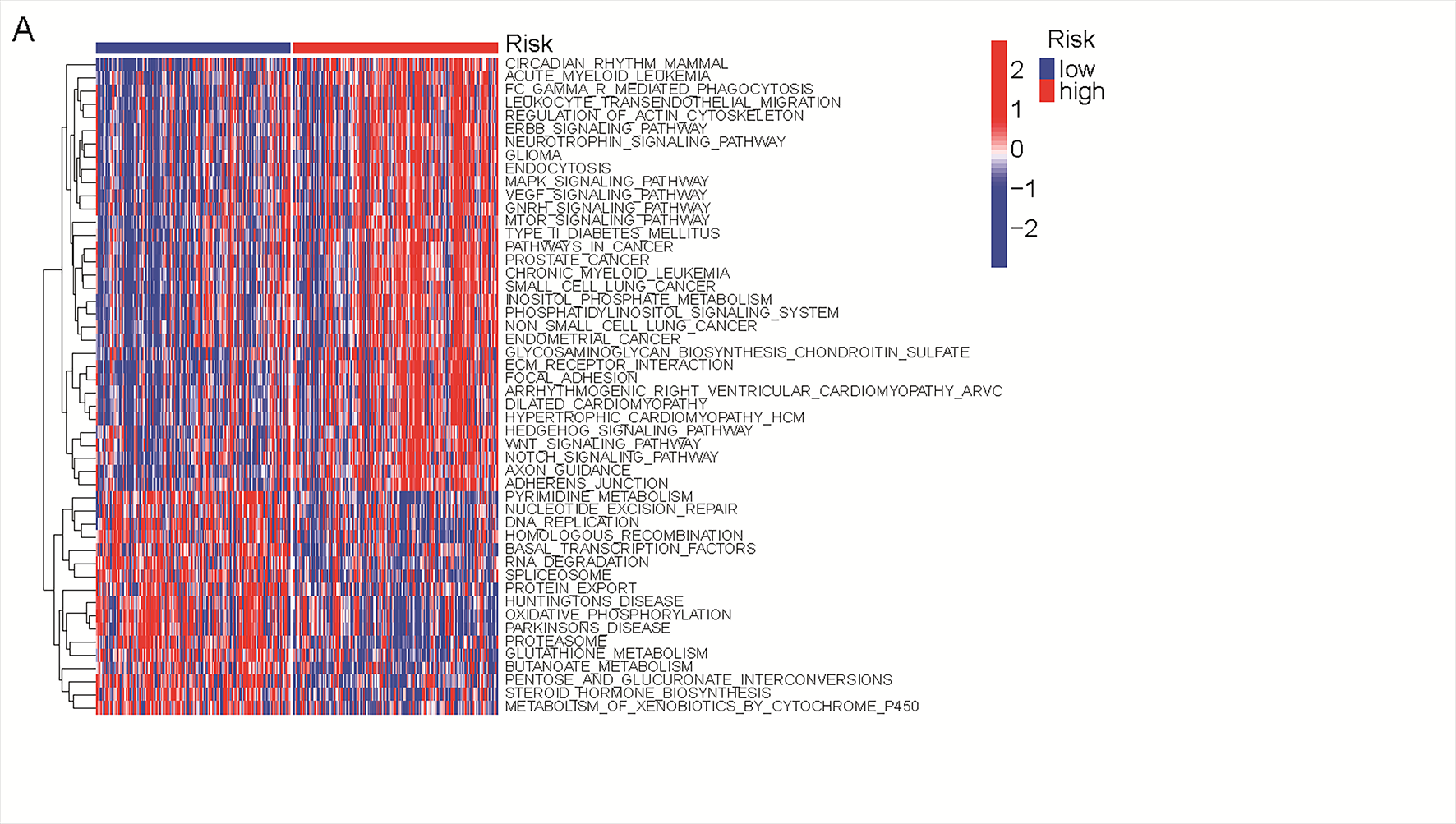

Supplement: Supplementary Figure 3 — Correlation analysis of risk score and infiltration of seven immune and stromal cells from MCP-COUNTER. Correlation analysis between B lineage (A), endothelial cells (B), fibroblasts (C), monocytic lineage (D), myeloid dendritic cells (E), neutrophils (F), and T cells (G). MCP-COUNTER: Microenvironment Cell Populations-counter. [file Image_3.tif]

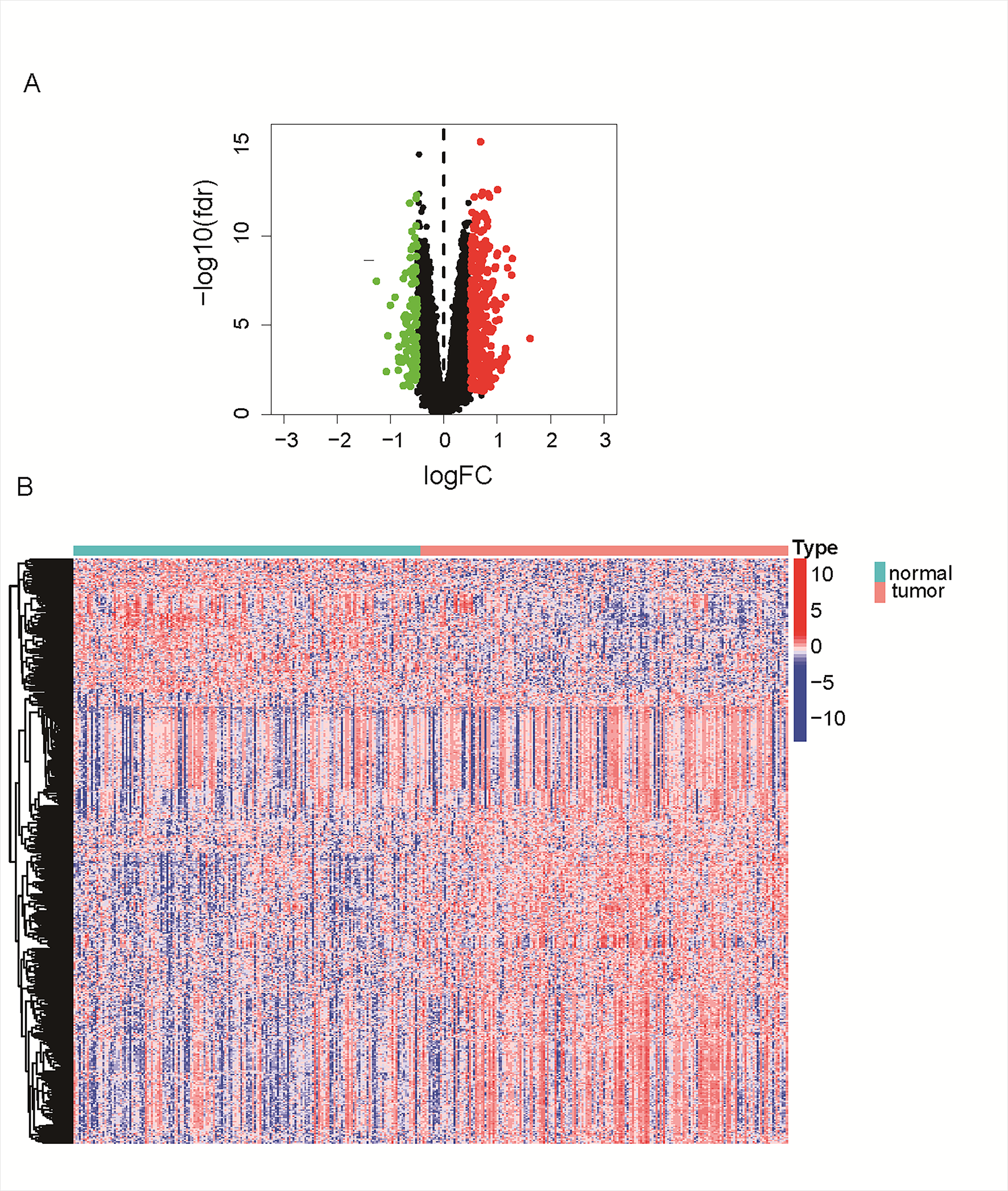

Supplement: Supplementary Figure 4 — Differences of immune-related functions among low- and high-risk groups in TCGA cohorts. TCGA: The Cancer Genome Atlas. [file Image_4.tif]

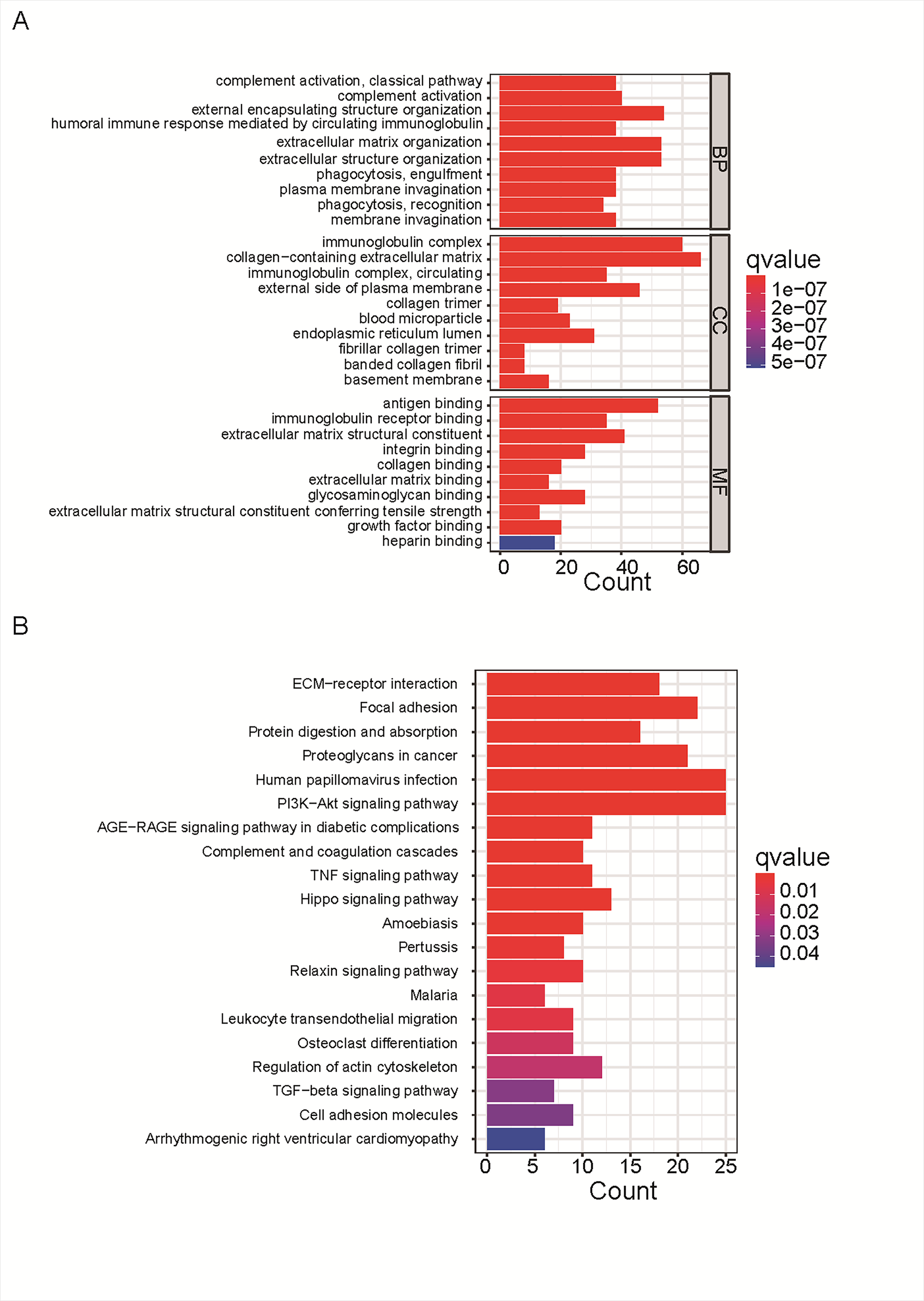

Supplement: Supplementary Figure 5 — Comparison of GSVA score between low-risk and high-risk groups. GSVA: gene set variation analysis. [file Image_5.tif]

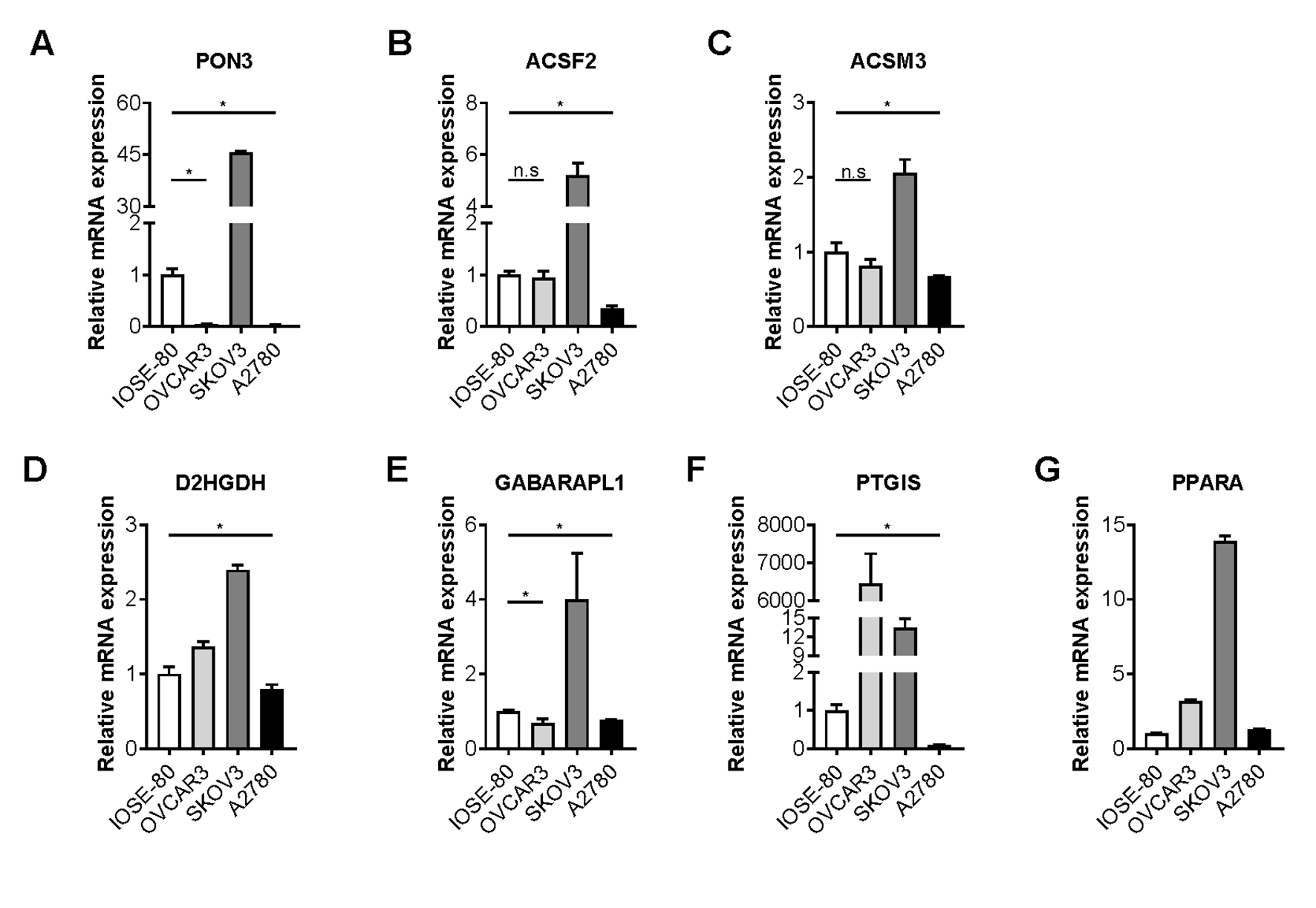

Supplement: Supplementary Figure 6 — Differentially expressed genes (DEGs) among low- and high-risk groups in TCGA cohorts. (A) Volcano plot for DEGs. Red pot represents DEGs with adjusted P < 0.05 and |logFC|≥0.5. (B) Heat map of the DEGs among low- and high-risk groups. The color red represents high-expression genes and color green represents low-expression genes. TCGA: The Cancer Genome Atlas; DEGs: differential expression genes; FC: fold change. [file Image_6.tif]

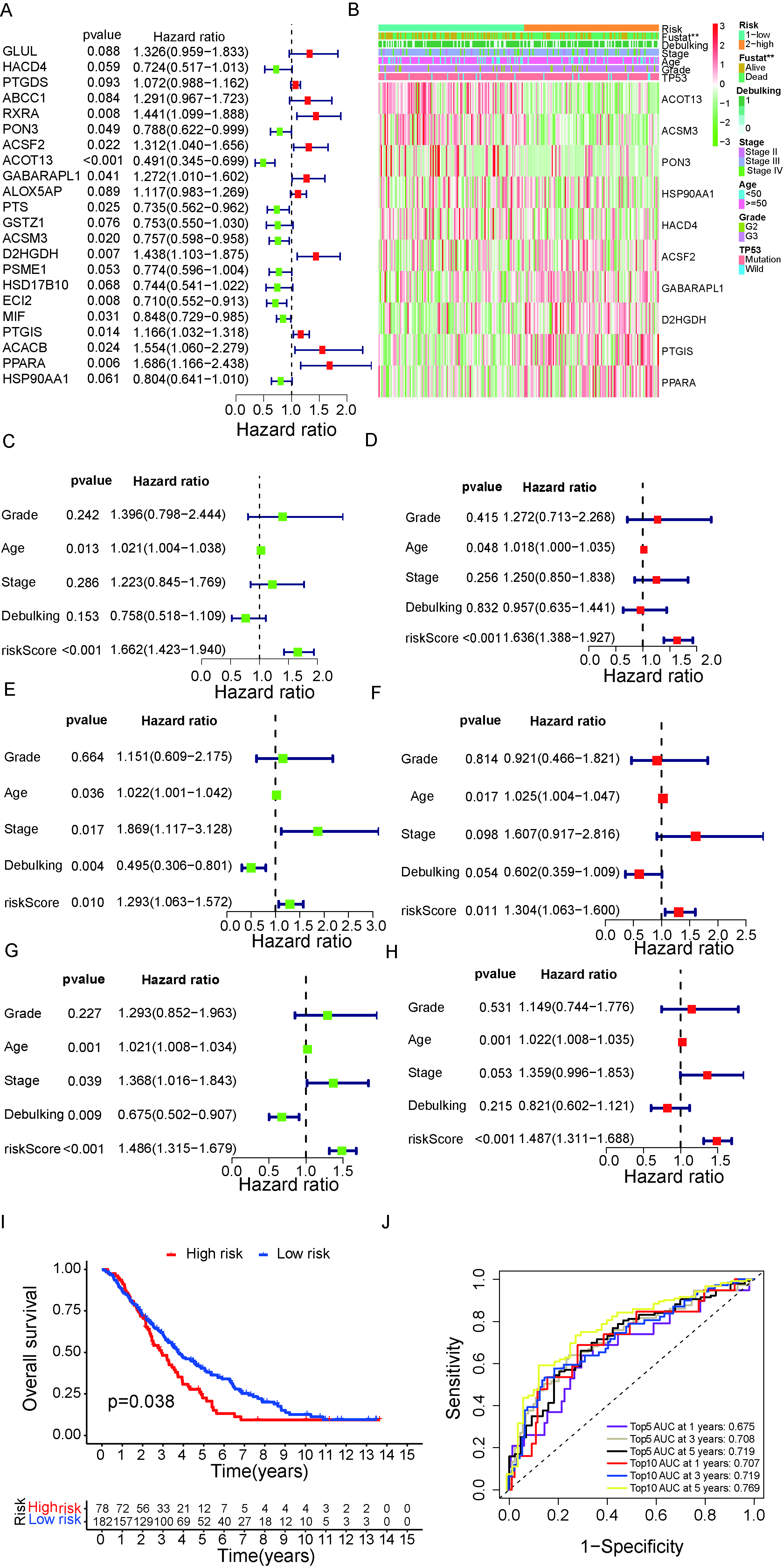

Supplement: Supplementary Figure 7 — Enrichment analysis of the DEGs between the low- and high-risk groups. (A) Bar plot of GO analysis. (B) Bar plot of KEGG analysis. DEG: differentially expressed genes; GO: gene ontology; KEGG: Kyoto encyclopedia of genes and genomes. [file Image_7.tif]

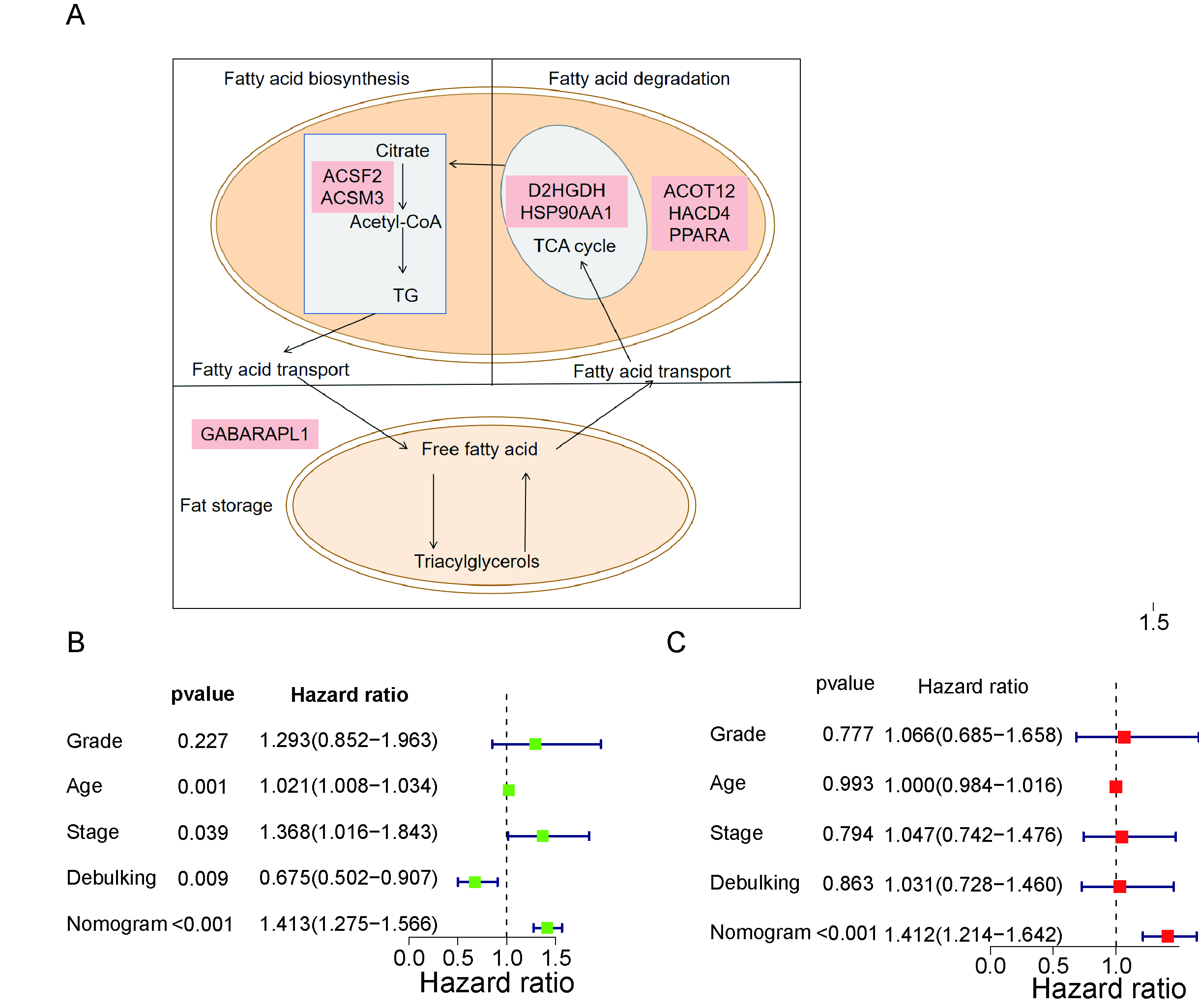

Supplement: Supplementary Figure 8 — Candidate genes expression in ovarian cancer cells. Gene expression of PON3 (A), ACSF2 (B), ACSM3 (C), D2HGDH (D), GABARAPL1 (E), PTGIS (F), and PPARA (G) in ovarian cancer cells (OVCAR3, SKOV3, and A2780 cells) versus normal ovarian cells (IOSE80 cells) determined by RT-qPCR analysis. RT-qPCR: reverse transcription and quantitative real-time polymerase chain reaction. [file Image_8.tif]
