# Supplementary material for: Identification of fatty acid signature to predict prognosis and guide clinical therapy in patients with ovarian cancer
Source: Front Oncol. 2022 Oct 4;12:979565. doi: 10.3389/fonc.2022.979565 (PMC9577003; doi:10.3389/fonc.2022.979565)
Supplement: Supplementary file 9 [file Table_1.docx]

**TableS1. Primers of ten candidate genes.**

| **Real-time PCR primer sequences for human genes** | | |
| --- | --- | --- |
| **Gene** | **Forward Primer** | **Reverse Primer** |
| HACD5 | 5’-TGTGGTGTGTGTTTTATTCGTC-3’ | 5’-TTGGTGGAATAAGTGCCAAATG-3’ |
| PON3 | 5’-TATGTTGTGAATCATCCCCACA-3’ | 5’-CAGGTATACCAGAGAACGTTGT-3’ |
| ACSF2 | 5’-CCGCTTCCTCAGTTCCAGAG-3’ | 5’-AGCTTTGTCCACCTCCTCCT-3’ |
| ACOT13 | 5’-TTTGACAGCCACGTTAGTAGAT-3’ | 5’-TTTCCTTGCTTCAGAACATGTG-3’ |
| GABARAPL1 | 5’-GAAGTGGGGCAGAAGGTGTT-3’ | 5’-ATAGCAGGAACTGCGATGGG-3’ |
| ACSM3 | 5’-TGAGGGCACTGCATAAAGATA-3’ | 5’-ATTTGAAGGTTTCTTTCCAGCC-3’ |
| D2HGDH | 5’-TTTACGTCCTCATCGAGACTTC-3’ | 5’-GAGGTCGTACTTGTACACGTAG-3’ |
| PTGIS | 5’-CTGATGTCTTCCACACCTTTCG-3’ | 5’-GACTTTTGACACTGCACATGT-3’ |
| PPARA | 5'-TTCGCCATGCTGTCTTCTGT-3’ | 5'-ACGTTTAGAAGGCCAGGACG-3’ |
| HSP90AA1 | 5'-CCAGTTCGGTGTTGGTTTTTAT-3’ | 5'-CAGTTTGGTCTTCTTTCAGGTG-3’ |
