# Supplementary material for: Identification of fatty acid signature to predict prognosis and guide clinical therapy in patients with ovarian cancer
Source: Front Oncol. 2022 Oct 4;12:979565. doi: 10.3389/fonc.2022.979565 (PMC9577003; doi:10.3389/fonc.2022.979565)
Supplement: Supplementary file 10 [file Table_2.docx]

**TableS2. Differentially expressed genes (DEGs) among normal tissue and tumor**

| **Gene** | **Mean-Normal** | **Mean-Tumor** | **logFC** | **pValue** | **fdr** |
| --- | --- | --- | --- | --- | --- |
| XIST | 7.533335799 | 1.650059901 | -5.883275898 | 2.05E-48 | 6.31E-47 |
| ACSM3 | 6.594089678 | 1.870167635 | -4.723922043 | 3.67E-48 | 6.86E-47 |
| ADH1B | 5.354256345 | 0.659551363 | -4.694704982 | 6.40E-46 | 4.30E-45 |
| ACAD11 | 4.106062246 | 0.114363149 | -3.991699097 | 2.05E-48 | 6.31E-47 |
| ALDH1A1 | 5.959926561 | 2.907903001 | -3.052023561 | 3.55E-45 | 1.90E-44 |
| ACADL | 3.077320859 | 0.111641324 | -2.965679535 | 2.32E-48 | 6.31E-47 |
| ACADVL | 7.97901289 | 5.111684046 | -2.867328844 | 5.89E-48 | 7.72E-47 |
| PON3 | 3.359869193 | 0.523526019 | -2.836343175 | 5.05E-46 | 3.48E-45 |
| MAOA | 4.140483406 | 1.344216013 | -2.796267393 | 4.55E-45 | 2.38E-44 |
| GABARAPL1 | 5.867352951 | 3.106854346 | -2.760498605 | 8.02E-47 | 6.01E-46 |
| ALDH2 | 5.620694253 | 2.911642571 | -2.709051683 | 7.23E-48 | 7.98E-47 |
| GLUL | 8.28844501 | 5.620280919 | -2.668164091 | 5.67E-48 | 7.72E-47 |
| PPT2 | 4.592893853 | 2.354232238 | -2.238661615 | 2.73E-48 | 6.31E-47 |
| ACACB | 3.356784934 | 1.132666069 | -2.224118865 | 5.18E-48 | 7.72E-47 |
| LTC4S | 2.411623224 | 0.283032032 | -2.128591192 | 6.43E-48 | 7.98E-47 |
| HSD17B4 | 5.333848702 | 3.279312541 | -2.054536161 | 2.14E-48 | 6.31E-47 |
| ACAA1 | 4.585892456 | 2.540106128 | -2.045786328 | 2.49E-48 | 6.31E-47 |
| SDHC | 5.35874831 | 3.319886323 | -2.038861988 | 2.05E-48 | 6.31E-47 |
| ACOX2 | 3.645631435 | 1.627957979 | -2.017673457 | 3.02E-45 | 1.68E-44 |
| EPHX2 | 4.226122778 | 2.328392025 | -1.897730753 | 8.71E-45 | 4.48E-44 |
| INMT | 2.536751863 | 0.677644498 | -1.859107365 | 4.94E-36 | 1.39E-35 |
| ACAT1 | 4.740218688 | 2.891901252 | -1.848317437 | 2.89E-48 | 6.31E-47 |
| ALDH7A1 | 5.542871598 | 3.741737248 | -1.80113435 | 6.91E-46 | 4.53E-45 |
| CPT1B | 2.299156364 | 0.543380931 | -1.755775433 | 5.32E-48 | 7.72E-47 |
| ECI2 | 4.804975638 | 3.175732053 | -1.629243584 | 3.07E-45 | 1.68E-44 |
| D2HGDH | 4.268427695 | 2.682497642 | -1.585930052 | 5.16E-42 | 2.02E-41 |
| ACSF2 | 4.518781864 | 2.987030541 | -1.531751323 | 5.97E-40 | 1.98E-39 |
| ACSF3 | 3.088048733 | 1.562359063 | -1.52568967 | 2.56E-48 | 6.31E-47 |
| ENO3 | 2.793061293 | 1.293650243 | -1.49941105 | 5.91E-44 | 2.67E-43 |
| GRHPR | 5.338023743 | 3.857971809 | -1.480051934 | 8.33E-48 | 8.39E-47 |
| PCCA | 3.267064019 | 1.828824935 | -1.438239083 | 5.15E-47 | 3.97E-46 |
| AOC3 | 2.677447345 | 1.242943497 | -1.434503848 | 3.05E-23 | 6.00E-23 |
| ACBD6 | 4.270235576 | 2.857594782 | -1.412640793 | 7.52E-48 | 7.98E-47 |
| CPT1C | 2.707712653 | 1.317687081 | -1.390025572 | 6.58E-30 | 1.53E-29 |
| ETFDH | 3.232484537 | 1.863189359 | -1.369295178 | 2.87E-47 | 2.59E-46 |
| HSD17B7 | 3.080830255 | 1.711571194 | -1.369259062 | 9.11E-46 | 5.68E-45 |
| GGT5 | 3.111196171 | 1.750371041 | -1.36082513 | 1.00E-24 | 2.03E-24 |
| ACBD4 | 3.708015008 | 2.361219161 | -1.346795847 | 1.23E-43 | 5.38E-43 |
| PTGIS | 3.939323629 | 2.603084642 | -1.336238987 | 8.74E-19 | 1.59E-18 |
| FADS1 | 3.542477405 | 2.226293517 | -1.316183888 | 2.08E-26 | 4.43E-26 |
| ACSS1 | 3.561617209 | 2.245785245 | -1.315831964 | 3.92E-35 | 1.08E-34 |
| CA4 | 1.672373537 | 0.368876535 | -1.303497002 | 1.16E-36 | 3.37E-36 |
| LTA4H | 5.394175342 | 4.103498273 | -1.290677069 | 1.39E-40 | 4.79E-40 |
| AKR1C3 | 2.937450993 | 1.658262513 | -1.27918848 | 1.74E-23 | 3.45E-23 |
| RXRA | 3.857290304 | 2.617224878 | -1.240065427 | 6.39E-41 | 2.32E-40 |
| PDHA1 | 5.449595424 | 4.263781356 | -1.185814068 | 2.37E-42 | 9.40E-42 |
| ADH1C | 1.476786921 | 0.309770631 | -1.167016291 | 3.02E-39 | 9.65E-39 |
| ADH5 | 5.517673617 | 4.363800972 | -1.153872644 | 1.24E-40 | 4.40E-40 |
| DECR1 | 4.772203739 | 3.665871133 | -1.106332606 | 6.75E-40 | 2.21E-39 |
| SLC22A5 | 2.331202278 | 1.251292371 | -1.079909907 | 2.32E-44 | 1.13E-43 |
| SDHA | 5.133570723 | 4.06805728 | -1.065513444 | 2.32E-40 | 7.88E-40 |
| ACOT9 | 3.718136259 | 2.672772627 | -1.045363632 | 2.21E-43 | 9.50E-43 |
| SLC27A3 | 4.097998864 | 3.085755467 | -1.012243396 | 1.51E-30 | 3.56E-30 |
| SCP2 | 4.965509353 | 3.990822121 | -0.974687232 | 4.27E-39 | 1.35E-38 |
| HACD1 | 2.018957346 | 1.053953145 | -0.965004201 | 8.22E-26 | 1.71E-25 |
| NUDT7 | 2.034755481 | 1.081677956 | -0.953077525 | 1.96E-37 | 5.84E-37 |
| SLC25A17 | 3.815224669 | 2.90384755 | -0.91137712 | 2.02E-36 | 5.77E-36 |
| SCD5 | 4.759993819 | 3.912101009 | -0.84789281 | 2.84E-13 | 4.38E-13 |
| UROD | 5.224913886 | 4.389356023 | -0.835557863 | 1.14E-33 | 2.89E-33 |
| AMACR | 1.58270474 | 0.765143133 | -0.817561607 | 3.97E-40 | 1.33E-39 |
| G0S2 | 4.031496284 | 3.223587837 | -0.807908446 | 6.73E-12 | 9.69E-12 |
| MCEE | 3.157568781 | 2.364343922 | -0.793224859 | 4.22E-34 | 1.13E-33 |
| ALAD | 4.075354229 | 3.288091028 | -0.787263201 | 2.00E-27 | 4.41E-27 |
| DECR2 | 2.994809771 | 2.211106394 | -0.783703377 | 2.37E-27 | 5.17E-27 |
| MLYCD | 1.560522692 | 0.788347931 | -0.772174761 | 8.33E-44 | 3.70E-43 |
| ADSL | 4.23603491 | 3.488030851 | -0.748004059 | 1.04E-33 | 2.68E-33 |
| ACAD10 | 2.980050265 | 2.26870612 | -0.711344146 | 1.10E-30 | 2.62E-30 |
| FMO1 | 1.708077085 | 1.001961134 | -0.706115951 | 2.07E-14 | 3.27E-14 |
| CROT | 2.33961441 | 1.647379457 | -0.692234953 | 4.48E-31 | 1.09E-30 |
| LGALS1 | 8.601521073 | 7.911267835 | -0.690253238 | 2.32E-09 | 3.07E-09 |
| ACADSB | 2.679283544 | 2.008217557 | -0.671065987 | 3.10E-25 | 6.34E-25 |
| PTS | 3.586041098 | 2.918539887 | -0.667501211 | 1.88E-20 | 3.51E-20 |
| CD36 | 1.464567016 | 0.829860069 | -0.634706948 | 8.44E-17 | 1.45E-16 |
| ACOT11 | 1.827330155 | 1.200456239 | -0.626873916 | 4.33E-22 | 8.29E-22 |
| CBR4 | 2.632873486 | 2.015644334 | -0.617229152 | 5.94E-26 | 1.24E-25 |
| ACAT2 | 2.887117926 | 2.2905017 | -0.596616226 | 1.12E-13 | 1.75E-13 |
| ME1 | 2.257098501 | 1.66693168 | -0.590166822 | 2.20E-16 | 3.75E-16 |
| PPARA | 2.183473228 | 1.595802224 | -0.587671004 | 5.33E-26 | 1.13E-25 |
| CEL | 1.901119406 | 1.334095125 | -0.567024281 | 2.53E-15 | 4.19E-15 |
| ACAA2 | 3.858684376 | 3.294699831 | -0.563984545 | 3.67E-17 | 6.36E-17 |
| NCAPH2 | 4.16165112 | 3.61629815 | -0.54535297 | 8.01E-21 | 1.51E-20 |
| CBR3 | 2.365326649 | 1.820088198 | -0.545238451 | 4.58E-09 | 5.99E-09 |
| PTGR2 | 2.117627093 | 1.578571977 | -0.539055117 | 1.77E-24 | 3.54E-24 |
| ACOX3 | 2.528036777 | 1.9971987 | -0.530838076 | 2.55E-17 | 4.48E-17 |
| GSTZ1 | 2.159812177 | 1.632189369 | -0.527622809 | 8.40E-16 | 1.40E-15 |
| ACO2 | 3.874615546 | 4.388076977 | 0.513461431 | 2.77E-16 | 4.69E-16 |
| HACD3 | 3.658587409 | 4.17367602 | 0.515088611 | 3.77E-15 | 6.13E-15 |
| UGDH | 3.133560537 | 3.655724998 | 0.522164461 | 2.67E-19 | 4.96E-19 |
| GPX4 | 6.794263327 | 7.318106338 | 0.52384301 | 1.47E-14 | 2.33E-14 |
| ALOX15B | 0.650778556 | 1.193793824 | 0.543015268 | 9.01E-11 | 1.26E-10 |
| HADHA | 5.164712501 | 5.715695224 | 0.550982723 | 6.87E-28 | 1.52E-27 |
| BMPR1B | 1.597814188 | 2.148888211 | 0.551074023 | 6.37E-10 | 8.64E-10 |
| CPT1A | 2.978523684 | 3.536562789 | 0.558039105 | 4.09E-16 | 6.86E-16 |
| ACOT7 | 2.476377001 | 3.043863527 | 0.567486525 | 3.49E-15 | 5.71E-15 |
| HACD4 | 1.127398933 | 1.69568887 | 0.568289937 | 8.22E-23 | 1.60E-22 |
| ALDH3A1 | 0.515200864 | 1.090589347 | 0.575388483 | 1.71E-11 | 2.43E-11 |
| MAPKAPK2 | 4.27884129 | 4.861602983 | 0.582761693 | 6.09E-18 | 1.08E-17 |
| SMS | 4.931971458 | 5.532027326 | 0.600055868 | 3.32E-15 | 5.47E-15 |
| PLA2G4A | 1.442997388 | 2.072907224 | 0.629909836 | 5.03E-09 | 6.56E-09 |
| TP53INP2 | 2.660742321 | 3.297627449 | 0.636885128 | 6.44E-13 | 9.59E-13 |
| FAAH | 2.269571762 | 2.908855589 | 0.639283827 | 2.80E-18 | 5.05E-18 |
| PECR | 1.754912468 | 2.396466707 | 0.64155424 | 1.19E-24 | 2.40E-24 |
| METAP1 | 2.954860444 | 3.6110264 | 0.656165957 | 4.05E-31 | 9.91E-31 |
| ACOX1 | 2.394703919 | 3.052077228 | 0.657373309 | 8.72E-30 | 2.00E-29 |
| CYP1B1 | 1.501090439 | 2.185920975 | 0.684830536 | 8.14E-09 | 1.05E-08 |
| THEM5 | 0.176316389 | 0.874706287 | 0.698389898 | 2.89E-37 | 8.50E-37 |
| CYP4F3 | 0.187062015 | 0.886923271 | 0.699861255 | 5.04E-23 | 9.86E-23 |
| SLC27A2 | 0.184864662 | 0.894188583 | 0.709323921 | 1.52E-36 | 4.39E-36 |
| ACSL5 | 2.538526843 | 3.279447757 | 0.740920914 | 1.79E-13 | 2.79E-13 |
| ACOT13 | 1.998930436 | 2.760162008 | 0.761231572 | 4.81E-34 | 1.27E-33 |
| SDHD | 4.851336419 | 5.628862183 | 0.777525764 | 2.99E-27 | 6.48E-27 |
| PCCB | 2.496071129 | 3.27526017 | 0.779189041 | 9.99E-31 | 2.40E-30 |
| SCD | 3.742098042 | 4.53027931 | 0.788181268 | 2.53E-09 | 3.34E-09 |
| MGLL | 1.627521306 | 2.41646482 | 0.788943514 | 4.80E-15 | 7.76E-15 |
| VNN1 | 0.211735247 | 1.01861001 | 0.806874763 | 2.61E-25 | 5.39E-25 |
| NDUFAB1 | 4.632978367 | 5.454444969 | 0.821466603 | 1.00E-33 | 2.60E-33 |
| HSP90AA1 | 6.862681765 | 7.684257463 | 0.821575697 | 9.87E-23 | 1.90E-22 |
| HPGD | 0.577247754 | 1.399842932 | 0.822595178 | 8.44E-06 | 1.02E-05 |
| OSTC | 4.920910347 | 5.750899747 | 0.8299894 | 4.21E-32 | 1.05E-31 |
| NBN | 2.597644768 | 3.437573142 | 0.839928373 | 2.55E-34 | 6.88E-34 |
| PSME1 | 6.271366139 | 7.118003465 | 0.846637326 | 4.56E-30 | 1.07E-29 |
| MCAT | 2.589444435 | 3.469603288 | 0.880158854 | 4.65E-39 | 1.45E-38 |
| FASN | 4.138626847 | 5.057363015 | 0.918736169 | 8.64E-19 | 1.58E-18 |
| TDO2 | 0.208832295 | 1.144195992 | 0.935363697 | 4.21E-28 | 9.42E-28 |
| ACBD5 | 1.790568634 | 2.733482251 | 0.942913618 | 3.08E-44 | 1.44E-43 |
| MIF | 6.854762495 | 7.823718996 | 0.968956501 | 3.13E-17 | 5.47E-17 |
| ACSL4 | 2.573051392 | 3.575194797 | 1.002143405 | 5.46E-29 | 1.24E-28 |
| SLC25A20 | 2.040546918 | 3.076148587 | 1.035601669 | 7.41E-38 | 2.23E-37 |
| ELOVL3 | 0.173472523 | 1.237243092 | 1.063770569 | 8.07E-36 | 2.25E-35 |
| SUCLG2 | 3.355692269 | 4.436606873 | 1.080914603 | 1.03E-41 | 3.90E-41 |
| ACLY | 3.292401392 | 4.384093913 | 1.091692521 | 6.56E-34 | 1.72E-33 |
| ABCC1 | 2.335207436 | 3.478619052 | 1.143411616 | 1.33E-40 | 4.63E-40 |
| HSD17B10 | 4.962917475 | 6.130310726 | 1.167393251 | 6.21E-38 | 1.89E-37 |
| LDHA | 6.154221271 | 7.377940766 | 1.223719495 | 2.04E-26 | 4.38E-26 |
| ALOX5 | 1.619934132 | 2.856464426 | 1.236530293 | 5.31E-21 | 1.01E-20 |
| CYP2J2 | 0.211638865 | 1.477785984 | 1.266147119 | 1.58E-42 | 6.48E-42 |
| EHHADH | 0.860330688 | 2.148101391 | 1.287770704 | 2.32E-45 | 1.32E-44 |
| ELOVL6 | 1.054919312 | 2.381206556 | 1.326287245 | 6.95E-40 | 2.25E-39 |
| PRDX6 | 6.358333267 | 7.691014729 | 1.332681462 | 3.72E-47 | 3.25E-46 |
| SLC25A1 | 4.339608233 | 5.677624722 | 1.338016489 | 2.34E-42 | 9.40E-42 |
| ACOT4 | 0.38898566 | 1.747453014 | 1.358467354 | 2.76E-44 | 1.32E-43 |
| FH | 3.571558232 | 4.965943903 | 1.394385671 | 2.27E-45 | 1.32E-44 |
| ALOX5AP | 2.297929329 | 3.704229372 | 1.406300043 | 5.30E-18 | 9.51E-18 |
| ELOVL7 | 0.335587781 | 1.746940271 | 1.411352489 | 6.28E-43 | 2.65E-42 |
| CPT2 | 1.915749338 | 3.332407523 | 1.416658185 | 2.83E-46 | 2.01E-45 |
| HCCS | 2.146110191 | 3.58778051 | 1.441670319 | 7.64E-46 | 4.88E-45 |
| THRSP | 0.113559273 | 1.606412744 | 1.492853471 | 9.23E-39 | 2.84E-38 |
| YWHAH | 4.464087203 | 5.962272925 | 1.498185723 | 6.00E-42 | 2.31E-41 |
| PTGES | 0.998250631 | 2.504636773 | 1.506386142 | 1.19E-28 | 2.68E-28 |
| MDH2 | 5.167377934 | 6.684936559 | 1.517558625 | 2.62E-47 | 2.46E-46 |
| ELOVL1 | 3.978943035 | 5.516227086 | 1.537284051 | 5.36E-44 | 2.46E-43 |
| CYP4F11 | 0.2361312 | 1.787855287 | 1.551724087 | 6.44E-35 | 1.76E-34 |
| ACBD7 | 0.187523757 | 1.754509008 | 1.566985251 | 7.41E-48 | 7.98E-47 |
| APEX1 | 5.820375306 | 7.392830152 | 1.572454846 | 4.95E-47 | 3.93E-46 |
| DPEP3 | 0.086755441 | 1.662624503 | 1.575869062 | 1.92E-08 | 2.45E-08 |
| ABCD1 | 1.75663728 | 3.422866827 | 1.666229547 | 2.03E-45 | 1.23E-44 |
| NSDHL | 2.430155455 | 4.130259278 | 1.700103823 | 1.34E-42 | 5.59E-42 |
| GPD2 | 1.980022615 | 3.690296676 | 1.710274061 | 7.61E-48 | 7.98E-47 |
| PTGDS | 3.873909524 | 5.589230528 | 1.715321004 | 3.09E-13 | 4.74E-13 |
| UBE2L6 | 4.395712855 | 6.1490158 | 1.753302944 | 8.23E-41 | 2.95E-40 |
| ODC1 | 4.409494514 | 6.192434859 | 1.782940345 | 5.41E-41 | 2.00E-40 |
| PPT1 | 4.266161205 | 6.05066561 | 1.784504404 | 1.81E-47 | 1.76E-46 |
| CA2 | 0.504035576 | 2.308183627 | 1.804148051 | 1.24E-44 | 6.13E-44 |
| ALDH1B1 | 2.166331206 | 3.978030377 | 1.811699171 | 3.23E-41 | 1.21E-40 |
| ECHS1 | 4.725934996 | 6.581016429 | 1.855081433 | 2.16E-48 | 6.31E-47 |
| HSD17B8 | 2.127430114 | 3.988242058 | 1.860811944 | 1.03E-44 | 5.19E-44 |
| HACD2 | 2.17851439 | 4.141467078 | 1.962952688 | 3.18E-48 | 6.41E-47 |
| FAAH2 | 0.623972988 | 2.597505085 | 1.973532097 | 4.71E-47 | 3.86E-46 |
| PCBD1 | 4.642121863 | 6.617934871 | 1.975813009 | 4.71E-47 | 3.86E-46 |
| PRXL2B | 2.180780065 | 4.176164023 | 1.995383957 | 1.03E-46 | 7.52E-46 |
| NUDT19 | 1.385870742 | 3.427701436 | 2.041830693 | 5.12E-48 | 7.72E-47 |
| CYP4B1 | 0.857307024 | 3.523053003 | 2.665745979 | 2.08E-31 | 5.13E-31 |
| GPX1 | 5.788796469 | 8.639547282 | 2.850750813 | 2.05E-48 | 6.31E-47 |
| DHCR24 | 3.32011379 | 7.276171492 | 3.956057701 | 2.20E-45 | 1.31E-44 |
| IL4I1 | 0.363055917 | 4.731592359 | 4.368536442 | 4.62E-48 | 7.72E-47 |
| PTGS1 | 0.926966323 | 5.621629902 | 4.694663579 | 2.80E-48 | 6.31E-47 |
