# Supplementary material for: Identification of fatty acid signature to predict prognosis and guide clinical therapy in patients with ovarian cancer
Source: Front Oncol. 2022 Oct 4;12:979565. doi: 10.3389/fonc.2022.979565 (PMC9577003; doi:10.3389/fonc.2022.979565)
Supplement: Supplementary file 11 [file Table_3.docx]

**TableS3. The corresponding regression coefficients of 10 final candidate genes.**

| **Gene** | **coefficient** |
| --- | --- |
| HACD4 | -0.40 |
| PON3 | -0.18 |
| ACSF2 | 0.22 |
| ACOT13 | -0.63 |
| GABARAPL1 | 0.24 |
| ACSM3 | -0.29 |
| D2HGDH | 0.33 |
| PTGIS | 0.10 |
| PPARA | 0.51 |
| HSP90AA1 | -0.22 |
