# Supplementary material for: Identification of fatty acid signature to predict prognosis and guide clinical therapy in patients with ovarian cancer
Source: Front Oncol. 2022 Oct 4;12:979565. doi: 10.3389/fonc.2022.979565 (PMC9577003; doi:10.3389/fonc.2022.979565)
Supplement: Supplementary file 12 [file Table_4.docx]

**TableS4. Drugs with significant different IC50 (diff P value<0.001) among low- and high-risk groups and the correlation analysis (cor P value<0.001) with risk score in TCGA cohort.**

| **Drug** | **diff P value** | **cor P value** | **cor** |
| --- | --- | --- | --- |
| Erlotinib | 2.61E-05 | 2.24E-09 | -0.305 |
| Sunitinib | 2.29E-06 | 7.92E-10 | -0.313 |
| AZ628 | 4.02E-05 | 1.84E-07 | -0.268 |
| Sorafenib | 1.48E-07 | 2.81E-10 | -0.321 |
| TAE684 | 3.06E-07 | 7.08E-11 | -0.331 |
| Saracatinib | 3.65E-10 | 1.41E-13 | -0.373 |
| Dasatinib | 5.12E-12 | 1.21E-16 | -0.414 |
| CGP-60474 | 0.000279067 | 2.83E-07 | -0.264 |
| CGP-082996 | 6.08E-08 | 1.61E-13 | -0.372 |
| A-770041 | 4.15E-07 | 6.42E-11 | -0.332 |
| WH-4-023 | 2.49E-08 | 2.05E-11 | -0.34 |
| WZ-1-84 | 7.44E-15 | 1.56E-24 | -0.499 |
| BMS-509744 | 4.21E-06 | 8.15E-09 | -0.295 |
| Pyrimethamine | 0.000378584 | 6.70E-06 | 0.232 |
| KIN001-135 | 3.28E-06 | 5.56E-06 | 0.234 |
| TGX221 | 1.48E-06 | 2.74E-10 | -0.321 |
| Bortezomib | 4.82E-06 | 5.98E-07 | -0.257 |
| XMD8-85 | 8.06E-13 | 7.36E-25 | -0.502 |
| Lapatinib | 2.23E-05 | 1.34E-10 | -0.327 |
| NSC-87877 | 0.00071698 | 0.000461419 | 0.182 |
| QS11 | 1.80E-05 | 6.01E-07 | 0.257 |
| Midostaurin | 3.29E-07 | 1.91E-08 | -0.288 |
| AP-24534 | 0.000223928 | 3.80E-06 | -0.238 |
| FH535 | 0.000374372 | 0.000259393 | 0.189 |
| PAC-1 | 3.60E-05 | 2.24E-07 | 0.266 |
| GSK-650394 | 0.00030163 | 0.00017573 | 0.194 |
| BMS-754807 | 0.000230934 | 6.97E-05 | -0.206 |
| Lisitinib | 1.11E-05 | 1.36E-06 | -0.249 |
| Bleomycin | 0.000727287 | 1.26E-06 | -0.249 |
| Bryostatin 1 | 1.38E-05 | 9.46E-05 | 0.202 |
| GSK1904529A | 0.000327739 | 5.18E-05 | 0.209 |
| TAK-715 | 9.01E-09 | 4.80E-10 | 0.317 |
| XL-184 | 3.91E-08 | 7.58E-11 | -0.331 |
| Zibotentan | 3.29E-05 | 1.10E-05 | 0.227 |
| YM155 | 2.40E-05 | 9.40E-06 | -0.229 |
| Linifanib | 9.69E-07 | 1.82E-10 | -0.324 |
| MP470 | 1.81E-10 | 9.47E-15 | 0.389 |
| SB52334 | 6.26E-06 | 7.49E-11 | 0.331 |
| Foretinib | 1.08E-08 | 8.55E-13 | -0.361 |
| CI-1040 | 0.000340933 | 1.07E-09 | -0.311 |
| AZD7762 | 0.000647382 | 2.77E-06 | -0.242 |
| 17-AAG | 5.28E-06 | 2.51E-10 | -0.322 |
| AMG-706 | 0.000194422 | 1.07E-05 | -0.227 |
| PD-0325901 | 2.55E-07 | 3.36E-15 | -0.395 |
| HG-5-113-01 | 0.000177749 | 4.85E-07 | -0.259 |
| HG-5-88-01 | 2.51E-14 | 9.62E-24 | -0.491 |
| TW 37 | 0.000797707 | 6.21E-05 | 0.207 |
| ZG-10 | 0.000127337 | 3.62E-06 | -0.239 |
| XMD8-92 | 4.16E-08 | 2.72E-12 | -0.354 |
| QL-VIII-58 | 0.000330215 | 3.23E-05 | -0.215 |
| QL-XII-61 | 0.000286587 | 3.53E-06 | -0.239 |
| PFI-1 | 4.10E-08 | 4.82E-11 | -0.334 |
| (5Z)-7-Oxozeaenol | 4.14E-07 | 3.94E-11 | -0.335 |
| FK866 | 1.31E-05 | 1.21E-06 | 0.25 |
| Trametinib | 5.02E-06 | 4.27E-10 | -0.318 |
| Temozolomide | 0.000665061 | 0.00058816 | 0.178 |
| TCGA, The Cancer Genome Atlas; IC50, half maximal inhibitory concentration. | | | |
